# Investigation of correlation between cholesterol intake, apolipoprotein B and Parkinson’s disease related genes in guinea pigs feeding a high-fat diet containing cholesterol

**DOI:** 10.1371/journal.pone.0352642

**Published:** 2026-06-25

**Authors:** Pinar Kacamak, Cigdem Elmas, Hatice Ayse Tokcaer Bora, Seniha Selcen Babaoglu Aydas

**Affiliations:** 1 Department of Histology and Embryology, Faculty of Medicine, Yozgat Bozok University, Yozgat, Turkey; 2 Department of Histology and Embryology, Faculty of Medicine, Gazi University, Ankara, Turkey; 3 Department of Neurology, Faculty of Medicine, Gazi University, Ankara, Turkey; 4 Vocational School of Health Services, Gazi University, Ankara, Turkey; University College London, UNITED KINGDOM OF GREAT BRITAIN AND NORTHERN IRELAND

## Abstract

Apolipoprotein B (Apo B), which is involved in the transport of cholesterol, is thought to be associated with neurodegenerative diseases such as Parkinson’s disease in addition to atherosclerosis and cardiovascular diseases. We aimed to investigate the possible correlation between cholesterol intake, Apo B and parkin RING domain-containing E3 ubiquitin protein ligase (PARKIN), phosphatase and tensin homologue (PTEN)-induced kinase 1 (PINK1) and α-synuclein (SNCA), which have an important role in Parkinson’s disease. Throughout the 12-week experiment, female and male guinea pigs in control group were fed a standard chow diet, while those in experimental group were fed a high-fat diet containing cholesterol. When histochemical findings were analysed at the end of our study, neuronal degeneration in the midbrain and brain cortex sections of the group of male guinea pigs fed a high-fat diet containing cholesterol was more pronounced compared to the other groups. In addition, significant differences were observed between the groups in terms of PARKIN expression levels (p = 0.030) in the brain tissues and the immunolabeling densities of PINK1 (p = 0.027), phospho(ser228)-PINK1 (p = 0.031), phospho(ser129)-SNCA (p < 0.000), and tyrosine hydroxylase (TH) (p = 0.033), particularly in the midbrain sections. Significant strong positive correlations (+0.5 < r<+1.0, p < 0.05) were observed in midbrain sections between phospho(Ser228)-PINK1 and TH immunolabeling and cholesterol (CHOL) levels, between phospho(Ser228)-PINK1 immunolabeling and low-density lipoprotein (LDL) levels, and between SNCA, phospho(Ser228)-PINK1, phospho(Ser129)-SNCA, and TH immunolabeling and high-density lipoprotein (HDL) levels. Our study demonstrated that a high-fat diet containing cholesterol was associated with significant changes in PARKIN gene expression and significant alterations in PINK1 protein levels in male guinea pigs in the experimental group.

## Introduction

Parkinson’s disease is the second most common neurodegenerative disorder after Alzheimer’s disease [1]. In epidemiological studies, it has been reported that incidence increases with age [2–4]. Parkinson’s disease is more common in men than in women, with ratios ranging from approximately 1.1:1–3:1 [5–8]. This may be attributed to the protective effects of estrogen in women [9]. Sex differences in Parkinson’s disease have also been identified in animal models [2].

Parkinson’s disease is a progressive neurodegenerative disorder characterised by the loss of dopamine-secreting dopaminergic neurons in the substantia nigra and accumulations of mostly α-synuclein (α-syn) called Lewy bodies [10,11]. Its main clinical symptoms are bradykinesia, rigidity, resting tremor and postural reflex disorder [12]. Currently, only symptomatic treatment is available for Parkinson’s disease, and since many different mechanisms play a role in the pathogenesis of this disease, neuroprotective treatments that would pause or reverse the degenerative process are still in the experimental phase [13]. Studies investigating familial Parkinson’s disease have found at least 17 autosomal dominant and autosomal recessive gene mutations responsible for variants of the disease [14]. These include SNCA, PARKIN, and PINK1 [14]. Familial forms of Parkinson’s disease and associated gene mutations account for approximately 10% of cases and have distinct clinical and pathological phenotypes [13]. However, it has been found that many of the neurodegeneration mechanisms in familial Parkinson’s disease overlap with those discovered in sporadic Parkinson’s disease and idiopathic Parkinson’s disease [13–16]. This suggests that Parkinson’s disease is a disease associated with both environmental and genetic factors.

Lipids, as water-insoluble molecules, are transported in the blood as protein-bound structures called lipoprotein particles. They consist of a hydrophobic core of triglycerides and cholesterol esters surrounded by a single layer of hydrophilic phospholipids containing embedded apolipoproteins [17,18]. Apo B is a component of chylomicron, very low-density lipoprotein (VLDL), intermediate-density lipoprotein (IDL), and low-density lipoprotein (LDL) [19]. It is thought that changes in lipid metabolism may play a role in the pathogenesis of neurodegenerative diseases such as Parkinson’s disease [20]. However, it is not yet clear whether there is a connection between neurodegenerative processes and parameters involved in lipid metabolism [20–25]. Some of the studies on this subject are human studies, others are animal studies using model systems or transgenic subjects [26–34]. In particular, studies with transgenic animals show that high levels of Apo B protein may be associated not only with atherosclerosis and cardiovascular diseases but also with neurodegenerative changes [33]. However, there are also studies that do not establish a link between Apo B protein and neurodegenerative changes and these studies are very few in both aspects [30,31]. Cholesterol is a precursor to steroid hormones such as testosterone and estrogen, which have protective effects and regulate various functions in the brain [35,36]. It has been reported that abnormal cholesterol metabolism in the brain is associated with many neurodegenerative disorders such as Parkinson’s disease, as the human brain has the highest cholesterol levels in the body and contains approximately 20% of the body’s total cholesterol [37–39]. However, data on serum lipid profile and consequently cholesterol levels in human and animal studies of Parkinson’s disease are also inconsistent [26–32]. Therefore, the aim of our study is to investigate the possible correlation between the PARKIN, PINK1 and SNCA proteins, which have important roles in Parkinson’s disease and are associated with cholesterol intake and the Apo B protein, which is involved in lipid metabolism.

In the literature, wild-type and transgenic animals used in animal studies examining the relationship between lipid metabolism and Parkinson’s disease are usually mice or rats. Differences in lipoprotein metabolism result in a different serum lipid profile between humans and wild-type mice [40,41]. Due to the low LDL/HDL ratio, wild-type mice are protected against hypercholesterolaemia and resistant to atherosclerosis [40,41]. Therefore, several transgenic mouse models have been established for animal studies of hyperlipidaemia and hypercholesterolaemia; however, the lipid profile of transgenic animals is still not completely equivalent to that of humans [41,42]. On the other hand, the number of studies using guinea pigs in Parkinson’s disease model studies in the literature is also very few; because model systems are generally created for monitoring and treating clinical symptoms; however, guinea pigs are not at the top of the list because they are characteristically timid and tend to hide, and because motor symptoms can be difficult to monitor. In addition, since these studies are old, it is not possible to obtain reliable data. However, non-transgenic guinea pigs largely mimic human lipoprotein and cholesterol metabolism and are being used to study the mechanisms underlying the relationship between Alzheimer’s disease, aging, and nutrition [43–46]. Unlike most wild-type or transgenic species used to study lipid metabolism, guinea pigs have been reported to carry most of their plasma cholesterol in LDL, similar to humans, and to exhibit aortic plaque accumulation when fed a high-cholesterol diet [43,46]. Therefore, guinea pigs are ideal animals to study the potential relationship between Parkinson’s disease and cholesterol and lipoprotein metabolism.

## Materials and methods

### Animals

A sample size analysis was performed using G*Power (α = 0.05, power = 0.80). Assuming a large effect size (f = 0.40), based on expectations from preclinical experimental animal studies in the absence of prior guinea pig–specific data, the estimated total sample size was approximately 76 animals. However, due to ethical considerations under the principles of reduction in animal research and practical limitations related to resource availability, the final sample size was limited to 24 guinea pigs.

In our study, 24 male and female Hartley albino guinea pigs (*Cavia porcellus*), aged 8–10 weeks and weighing 300–350 g were obtained from Kobay Deney Hayvanları Laboratory. The animals were kept in quarantine at Gazi University Laboratory Animals Breeding and Experimental Researches Centre (GUDAM) for a week. During the quarantine process, one of the male guinea pigs died for unknown reasons and was excluded from the study. Consequently, all experimental procedures were carried out at GUDAM using the remaining 23 guinea pigs. The animals were randomly divided into 2 groups as 11 guinea pigs (6 females, 5 males) in the control group and 12 guinea pigs (6 females, 6 males) in the experimental group: Control Group (CG) and Experimental Group (EG). Then, the guinea pigs in the control group were divided into subgroups according to their sex as Control Female (CF) (n = 6 females) and Control Male (CM) (n = 5 males). The guinea pigs in the experimental group were divided into subgroups according to their sex as Experimental Female (EF) (n = 6 females) and Experimental Male (EM) (n = 6 males). During the 12-week experiment, the guinea pigs were kept in separate cages (2–3 guinea pigs from the same subgroup in one cage), in a 12 h light-dark cycle and at 20 ± 2 °C and the guinea pigs in the control group were given standard chow diet and water *ad libitum*, while the guinea pigs in the experimental group were given high-fat diet containing cholesterol and water *ad libitum* [47–51]. The standard chow diet consisted of 3% fat, 16% crude protein, 12.5% crude fiber, 10% crude ash, while the cholesterol-containing high-fat diet consisted of 15.5% fat, 22% crude protein, 12% cellulose, 41.3% carbohydrate, 1.5% mineral mix, 1% vitamin mix, 0.33% cholesterol [43,52–55]. In addition, the fat mix of the cholesterol-containing high-fat diet contained olive oil: palm kernel oil: safflower oil in the ratios of 1: 2: 1.8, respectively. Both diets were prepared by ‘ARDEN Araştırma & Deney’. Since neither of the two diets contained vitamin C, 1000 mg/1 L vitamin C was added to the daily changed water of both the control group and the experimental group [56]. Furthermore, small and equal amounts of fresh, washed carrot or iceberg lettuce were given to both the control and experimental groups 1–2 times a week for vitamin C supplementation [57]. After random grouping at the beginning of the experiment, each guinea pig was weighed. The guinea pigs were weighed once a week at the same time (between 11:00 a.m.-13:00 p.m.) throughout the experiment and again at the same time (between 11:00 a.m. - 13:00 p.m.) 1 day before sacrifice. Additionally, feed was weighed at the same time (between 11:00 a.m.-13:00 p.m.) every day throughout the experiment for routine health monitoring; however, since the daily feed amount consumed by the guinea pigs could not be determined due to the cage type, the data obtained were not evaluated statistically. It was unanimously approved that our study was in accordance with the principles of Gazi University Animal Experiments Local Ethics Committee (Document date and number: 03/01/2024-E.841869).

### Collection and storage of tissue samples

At the end of 12 weeks, each guinea pig was anaesthetised with sodium thiopental (100 mg/kg i.p.), placed in the supine position and the guinea pigs were sacrificed by sampling intracardiac blood under deep anaesthesia [47]. Blood samples obtained by cardiac puncture and collected in serum tubes were centrifuged at 1000–2000 x g for 15–20 min at 2–8 °C, the supernatants were transferred to microcentrifuge tubes and stored at −80 °C until ELISA and biochemical analysis. The brain and cerebellum tissues obtained were divided into two halves in the sagittal plane exactly in the middle, and then one half was dissected for RT-qPCR and ELISA, frozen in liquid nitrogen and stored at −80 °C until analysis, while the other half was placed in 4% paraformaldehyde fixative for histochemical and immunohistochemical analyses. After the optimum time, the tissues in the fixative were subjected to a light microscope tissue tracking process and embedded in paraffin.

### Histochemical analysis

4 micron (μ) thick sections were taken from the prepared paraffin blocks and stained with haematoxylin-eosin (H&E) and neutral red stain. The sections were photographed with Leica DM 4000 (Germany) computer assisted imaging system using Leica LAS V4.9 programme. The brain cortex, midbrain, and cerebellum sections of each subject were stained with H&E and neutral red; H&E staining was used to assess general neuronal morphology (e.g., neuronal degeneration, pyknotic nuclei and perineuronal vacuolization), while neutral red staining was used to evaluate neuronal characteristics, including cells with euchromatic nuclei and intensely stained neurons [58–62].

### Immunohistochemical analysis

Immunolabeling for PARKIN, PINK1, SNCA, low-density lipoprotein receptor (LDLR), phospho(ser65)-PARKIN, phospho(ser228)-PINK1, phospho(ser129)-SNCA, and TH was performed on the midbrain, brain cortex and cerebellum sections. Following deparaffinization and rehydration, a retrieval procedure was performed using citrate buffer (pH: 6.0). Then, endogenous peroxidase activity was blocked in the sections incubated with 3% hydrogen peroxide. The sections were incubated with UltraV block (Cat: TP-060-HL, Lot: PHL945, UltraVision Detection System Anti-Polyvalent, HRP (RTU), Thermo Scientific, USA) to prevent non-specific binding. The sections were incubated with PARKIN (Cat: PA1–18265, Invitrogen, USA), PINK1 (Cat: orb331223, Biorbyt, UK), SNCA (α-synuclein) (Cat: bs-0009R, Bioss Antibodies, USA), LDLR (Cat: bs-0705R, Bioss Antibodies, USA), phospho(ser65)-PARKIN (Cat: orb312554, Biorbyt, UK), phospho(ser228)-PINK1 (Cat: PA5–105356, Invitrogen, USA), phospho(ser129)-alpha-synuclein (Cat: PA5–104885, Invitrogen, USA) and tyrosine hydroxylase (Cat: bs-0016R, Bioss Antibodies, USA) primary antibodies at 4 °C for overnight. Dilution ratios of primary antibodies diluted with Large Volume UltrA Diluent (Cat: TA-125-UD, Lot: UD51340, Thermo Scientific, USA) are given in S1 Table. Then, the sections were incubated with biotinylated anti-polyvalent (Cat: TP-060-HL, Lot: PHL945, UltraVision Detection System Anti-Polyvalent, HRP (RTU), Thermo Scientific, USA). The sections were incubated with streptavidin peroxidase (Cat: TP-060-HL, Lot: PHL945, UltraVision Detection System Anti-Polyvalent, HRP (RTU), Thermo Scientific, USA). Finally, the sections were incubated with aminoethyl carbazole (AEC) chromogen (Cat: TA-125-HAS, Lot: HAS61420, UltraVision Detection System Large Volume AEC Substrate System (RTU), Thermo Scientific, USA) to ensure visible immune reaction. Mayer’s hematoxylin was used for conterstaining of the sections. After the dehydration step, all the sections were mounted with the water-based mounting medium. All steps except the stage, in which the primary antibodies were used, were performed at room temperature. The sections were photographed with Leica DM 4000 (Germany) computer assisted imaging system using Leica LAS V4.9 programme. Six random areas were selected at 400X magnification from each section prepared by immunohistochemical labeling. The density of immunopositive areas was determined as a percentage using the ImageJ software (National Institutes of Health, Bethesda, Maryland, USA). At the end of the evaluations, statistical data were generated for the immunohistochemical density of each primary antibody.

### Biochemical analysis

Measurement of CHOL, glucose (GLU), HDL and LDL levels in the serum samples was performed by Diagen Biotechnological Systems Inc. At the end of the measurements, statistical data was generated for each parameter.

### ELISA

**Fig 1:**
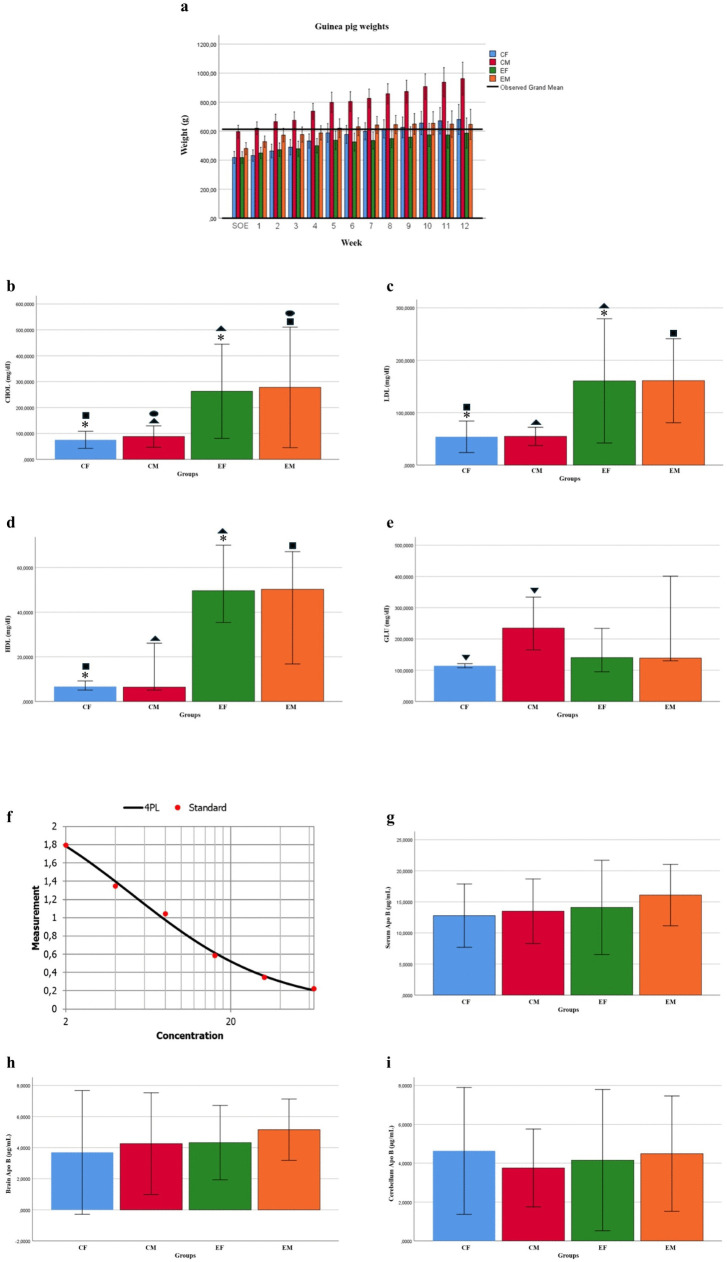
Graphs of weight measurement, biochemistry analysis and ELISA data. **(a)** An increase was observed in the body weights of both control and experimental groups from the beginning to the end of the experiment (p < 0.000, Repeated Measures ANOVA); however, this increase was found to be statistically significant in the CM group compared to the CF (p < 0.000, Tukey HSD), EF (p < 0.000, Tukey HSD) and EM (p = 0.002, Tukey HSD) groups (SOE: start of experiment). **(b-e)** When multiple comparisons were made between the groups, the difference between the groups in terms of CHOL (p < 0.000, ANOVA), LDL (p < 0.000, ANOVA), HDL (p = 0.001, Kruskal-Wallis test) and GLU (p = 0.006, Kruskal-Wallis test) levels in the serum samples was found to be statistically significant. It was determined that this situation was due to significant differences between CF and EF groups (*: CHOL (p = 0.002, Tukey HSD), LDL (p = 0.001, Tukey HSD), HDL (p = 0.012, Kruskal-Wallis test)), CF and EM groups (■ : CHOL (p = 0.001, Tukey HSD), LDL (p = 0.001, Tukey HSD), HDL (p = 0.028, Kruskal-Wallis test)), CM and EF groups (▲ : CHOL (p = 0.007, Tukey HSD, LDL (p = 0.001, Tukey HSD), HDL (p = 0.040, Kruskal-Wallis test)), CM and EM groups (● : CHOL (p = 0.003, Tukey HSD), LDL (p = 0.001, Tukey HSD)) and CF and CM groups (**▼** : GLU (p = 0.004, Kruskal-Wallis test). **(f)** Standard curve graph created for calculating ELISA results. **(g-i)** When multiple comparisons were made between the groups, there was no statistically significant difference between the groups in terms of Apo B levels in the serum (p = 0,271, ANOVA), brain (p = 0,427, ANOVA) and cerebellum (p = 0,790, ANOVA) tissues.

Guinea Pig Apolipoprotein B ELISA Kit (BT LAB: Bioassay Technology Laboratory, Catalog no: EA0004Gp, China) was used to measure Apo B levels in the serum, brain and cerebellum tissues obtained from guinea pigs. Homogenization of the brain and cerebellum tissues was performed in according to the kit protocol. All reagents, samples and standards were prepared in according to the kit protocol and all reagents were brought to room temperature before use. The optical density (OD value) of the plate was measured at 450 nm with a microplate reader. Calculation of results in ELISA is of great importance for the success of the test. Thus, the absorbance values for each standard and sample set were averaged and the standard curve in Fig 1 was created. The concentration of Apo B was calculated from the standard curve. As a result of the calculations, statistical data was created.

### RT qPCR

TRIzol Reagent was used to obtain high quality total RNA extraction. The tissue suspensions were physically disrupted using the FP120 FASTPREP (Thermo, USA) homogenizer device. RNA measurements of the tissues were performed using the Colibri+ Microvolume Spektro (Titertek Berthold, Germany) device. RNA loads (ng values) of the tissues obtained from the total RNA extraction process were fixed to a certain ng value to be used in the next steps of the study. Total cDNA was synthesized from RNA obtained from the brain and cerebellum tissues. For this, reverse transcription was performed using the SensiFASTTM cDNA synthesis kit containing 5x TransAmp Buffer and Reverse Transcriptase in accordance with the kit protocol. The components in the kit used for the reverse transcription process, which was performed using the protocol given in S2 Table in the ABI Veriti 96 Thermal Cycler (Applied Biosystems, USA), were prepared on ice and in a sterile tube. In the last stage, cDNA chains generated from mRNA sequences were used. RSP-16 was selected as the housekeeping or reference gene. To determine the mRNA expression levels of PARKIN, PINK1, SNCA, LDLR and RSP-16 genes, gene-specific primers were designed using the internet-based Primer3Plus (https://www.primer3plus.com/index.html) and validated via Integrated DNA Technologies (https://www.idtdna.com/pages/tools/oligoanalyzer). The specific primer list for the selected genes is given in S3 Table. The primers used were supplied by Oligomer. SensiFASTTM SYBR No-ROX Kit was used to determine the expression levels of RPS-16, PARKIN, PINK1, SNCA and LDLR. Thus; the amplification of the relevant mRNAs and the sequences of the appropriate reference gene for each of the brain and cerebellum tissues in the control and experimental groups using the Biorad CFX96 Touch real-time PCR device was achieved by adjusting the protocol given in S4 Table. At the end of the process, the data obtained through the device were evaluated with the 2^-(ΔΔCt)^ method [63]. In the 40-cycle study, the threshold value (Ct (threshold cycle), Cp (crossing point), Cq (quantification cycle)) taken from the point where the fluorescence value crossed the threshold value (crossing the threshold line) was used in the calculations. As a result of the calculations, statistical data was created.

### Statistical analysis

Statistical analyses were performed using SPSS 26.0 (IBM SPSS Statistics, USA). Data normality was assessed prior to analysis. For comparisons between independent groups, the independent samples t-test was used for parametric data, while the Mann–Whitney test was used for nonparametric data. One-way analysis of variance (ANOVA) followed by Tukey’s HSD post hoc test was used for multiple group comparisons of parametric variables. For nonparametric multiple group comparisons, the Kruskal–Wallis test was applied followed by Bonferroni-adjusted post hoc pairwise comparisons. Repeated Measures ANOVA was used for analysis of body weight data across time points. Correlation analyses were performed using Pearson’s correlation coefficient for parametric variables and Spearman’s rank correlation for nonparametric variables. Appropriate statistical corrections for multiple comparisons (Tukey or Bonferroni) were applied depending on the analysis performed. A p-value ≤ 0.05 was considered statistically significant.

## Results

### Body weights of guinea pigs

As a result of Mauchly’s sphericity test performed within the scope of repeated measures ANOVA, p < 0.000 was found (see S5 Table). Since a statistically significant difference was found with Mauchly’s sphericity test, pairwise comparisons and multiple comparisons were made. The p values for the pairwise and multiple comparisons are given in S6, S7 and S8 Tables. In addition, the graph for comparing the guinea pig weights by weeks and groups is given in Fig 1. In line with the results obtained, an increase was observed in the body weights of both the control (CG) and experimental (EG) groups from the beginning of the experiment to the end of the experiment; however, it was found that this increase was more consistent in the CM group and statistically significant compared with the CF, EF and EM groups (p < 0.000, p < 0.000 and p = 0.002, respectively). Although no statistically significant difference was found, it was noteworthy that the increase in the body weights of the female guinea pigs in the CF group was greater than the body weights of the female and male guinea pigs in the EF and EM groups.

### Histochemical results

**Fig 2:**
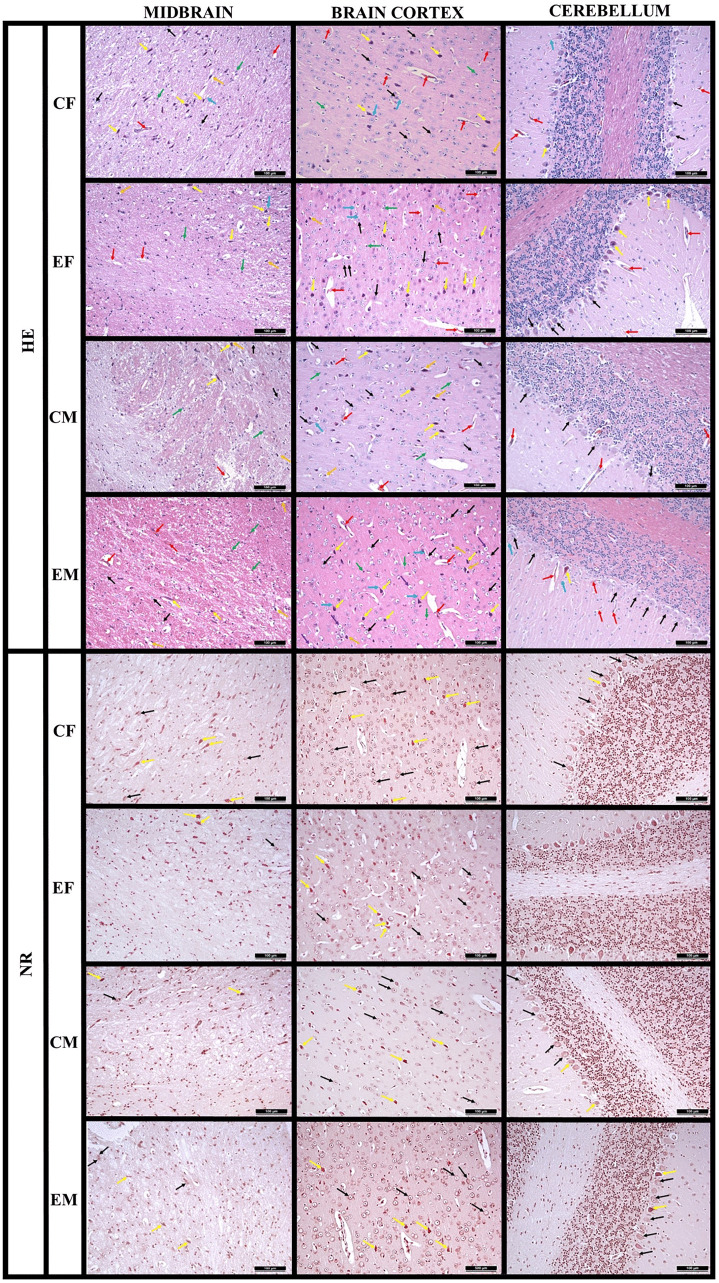
Histochemical findings in the midbrain, brain cortex and cerebellum sections of CF, EF, CM and EM groups. Black arrows in the midbrain and brain cortex sections: Neurons. Black arrows in the cerebellum section: Purkinje cells. Blue arrows: Neuron processes. Yellow arrows: Intensely stained neurons. Green arrows: Neuroglia cells. Orange arrows: Perineuronal vacuolisation. Purple arrows: Nuclear swelling. Red arrows: Blood vessels. HE: Hematoxylin-Eosin. NR: Neutral Red. The magnification of all micrographs is 200x.

The evaluations of H&E stained the midbrain and brain cortex sections of the EF group were similar to the control groups. Perineuronal vacuolisation in the midbrain sections and nuclear swelling in the brain cortex sections of the EM group were more prominent than the other groups. The evaluations of the H&E stained the cerebellum sections of the EF and EM groups were similar to those of the control groups. The findings obtained as a result of neutral red staining, which we applied to better detect both neurons with euchromatic nuclei and intensely stained neurons with pyknotic nuclei, supported the findings obtained as a result of H&E staining. Photographs showing H&E and neutral red staining of the midbrain, brain cortex and cerebellum sections of the groups are given in Fig 2.

### Immunohistochemical results

**Fig 3:**
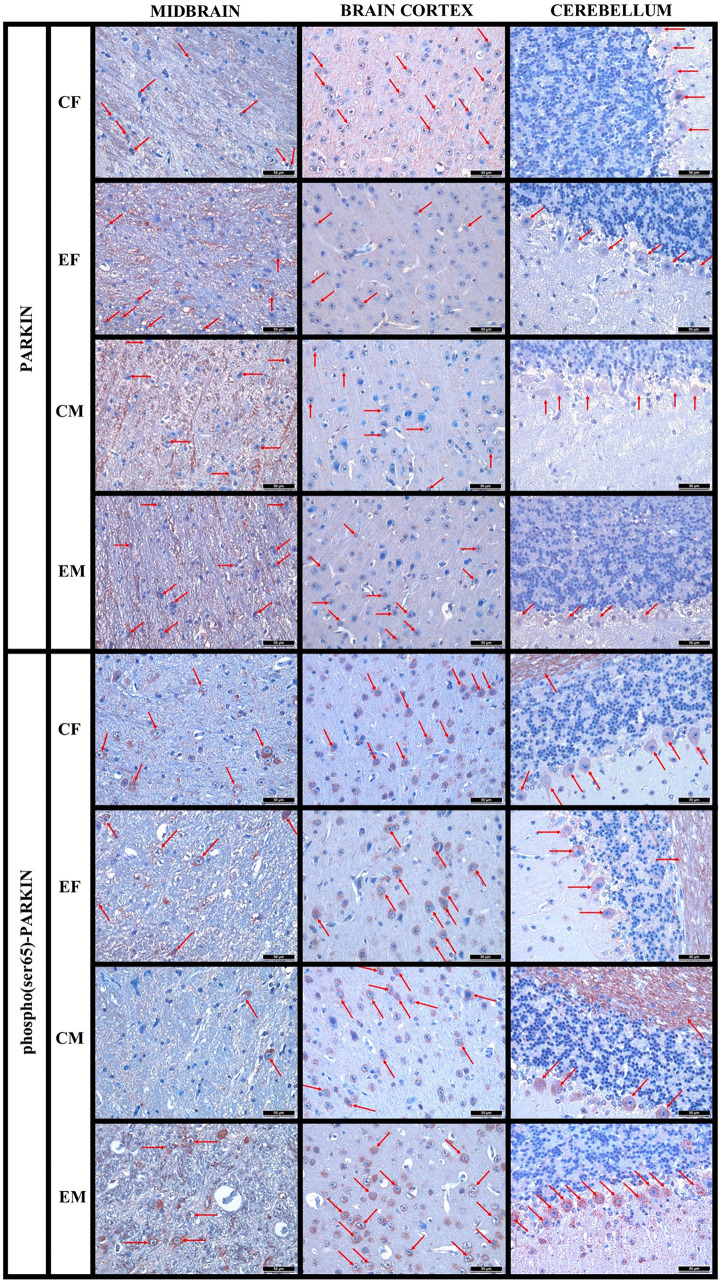
Immunohistochemical findings of PARKIN and phospho(ser65)-PARKIN in the midbrain, brain cortex and cerebellum sections of CF, EF, CM and EM groups. Red arrows: Immunopositive neurons. The magnification of all micrographs is 400x.

**Fig 4:**
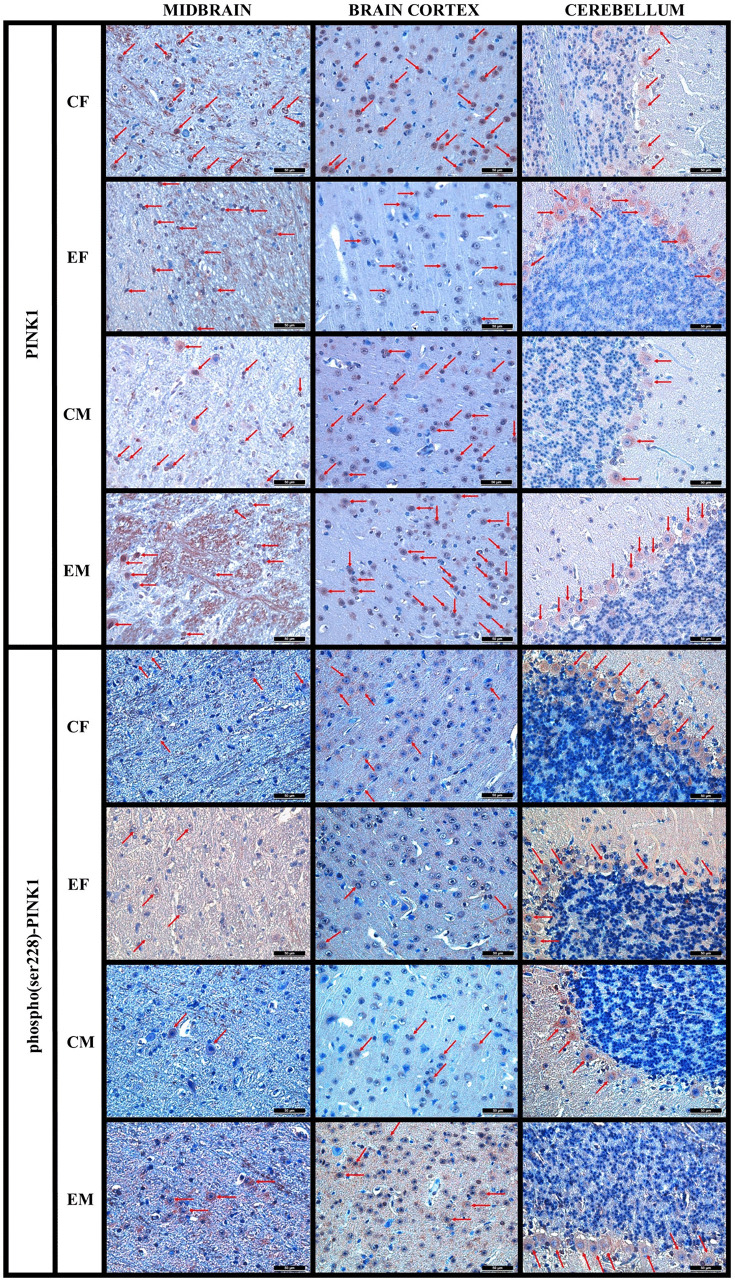
Immunohistochemical findings of PINK1 and phospho(ser228)-PINK1 in the midbrain, brain cortex and cerebellum sections of CF, EF, CM and EM groups. Red arrows: Immunopositive neurons. The magnification of all micrographs is 400x.

**Fig 5:**
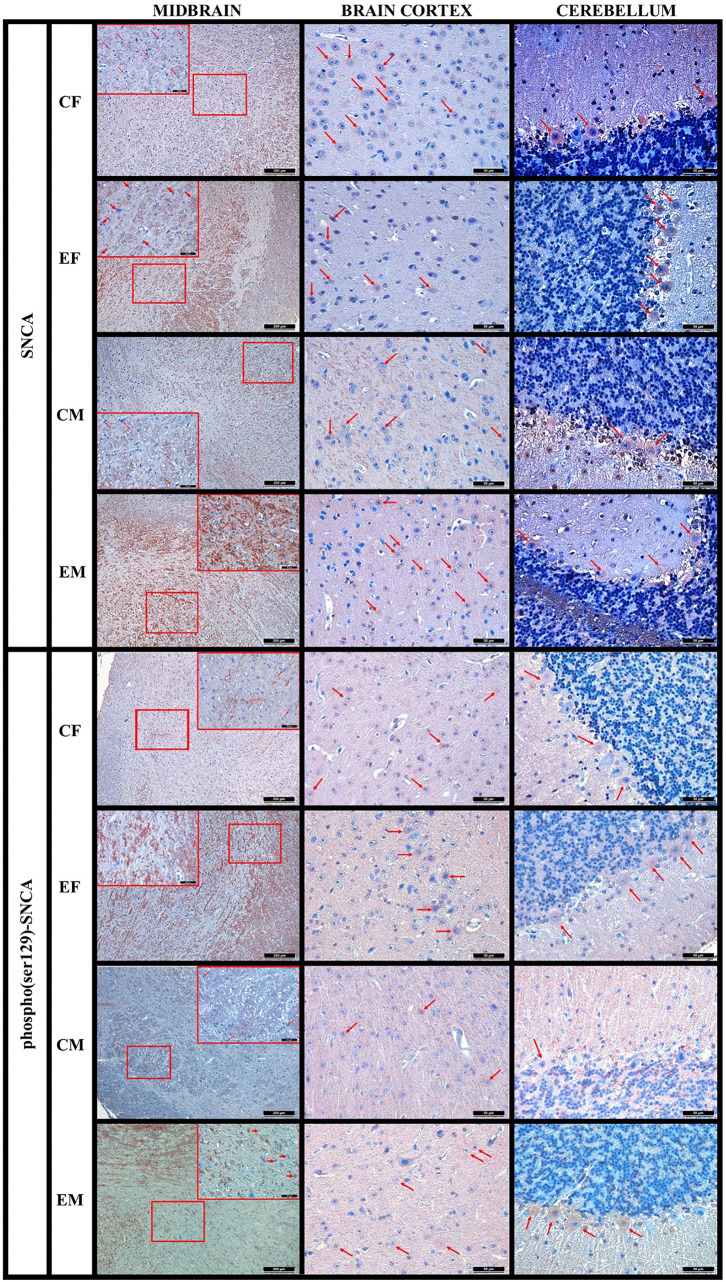
Immunohistochemical findings of SNCA and phospho(ser129)-SNCA in the midbrain, brain cortex and cerebellum sections of CF, EF, CM and EM groups. Red arrows: Immunopositive neurons. The magnification of small micrographs is 400x and the magnification of large micrographs is 100x.

**Fig 6:**
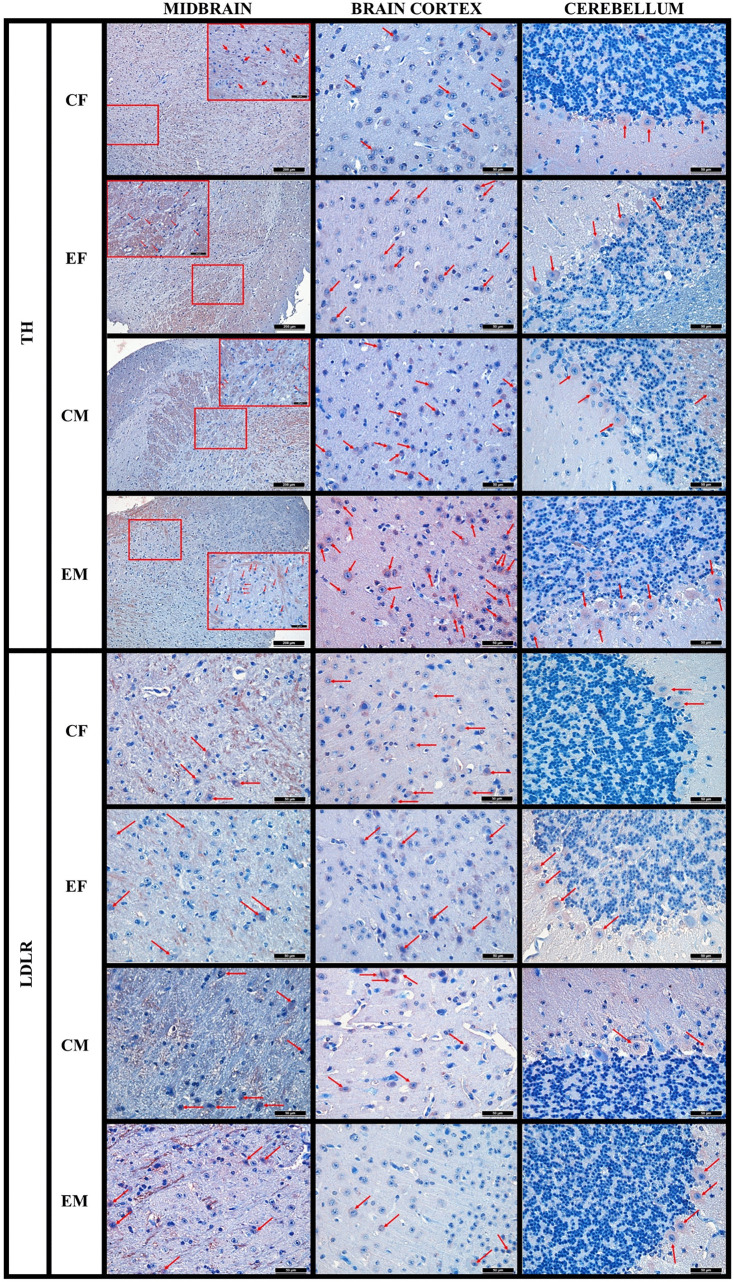
Immunohistochemical findings of TH and LDLR in the midbrain, brain cortex and cerebellum sections of CF, EF, CM and EM groups. Red arrows: Immunopositive neurons. The magnification of small micrographs is 400x and the magnification of large micrographs is 100x.

**Fig 7:**
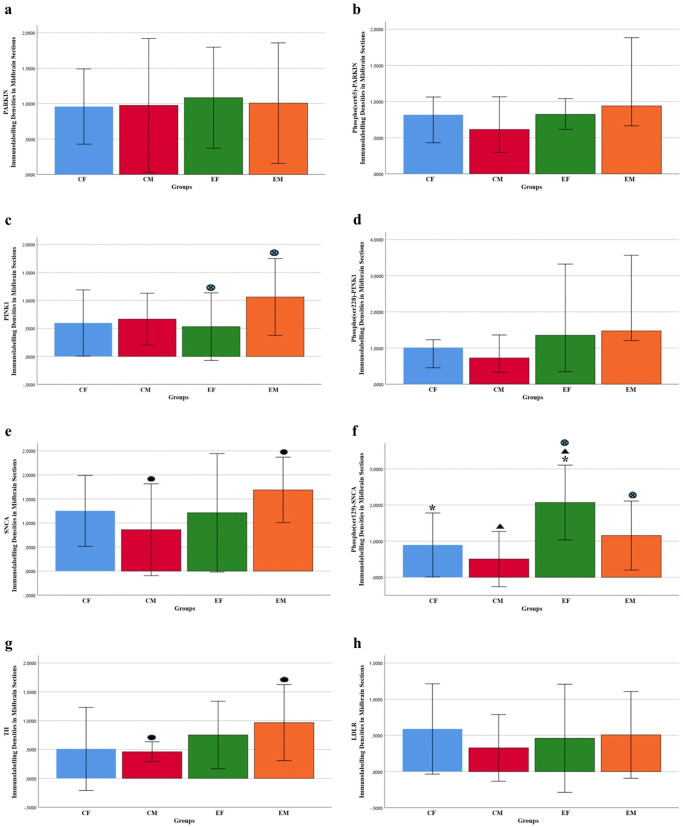
Graphs of multiple comparisons between groups of PARKIN, phospho(ser65)-PARKIN, PINK1, phospho(ser228)-PINK1, SNCA, phospho(ser129)-SNCA, TH and LDLR immunolabeling densities in the midbrain sections. **(a, b, h)** The difference between the groups in terms of PARKIN (p = 0.945, ANOVA), phospho(ser65)-PARKIN (p = 0.188, Kruskal-Wallis test) and LDLR (p = 0.582, ANOVA) immunolabeling densities in the midbrain sections was not statistically significant. **(c)** The difference between the groups in terms of PINK1 (p = 0.027, ANOVA) immunolabeling densities in the midbrain sections was found to be statistically significant and it was determined that this situation was due to significant differences between EF and EM groups (**: p = 0.029, Tukey HSD). **(d)** The difference between the groups in terms of phospho(ser228)-PINK1 (p = 0.031, Kruskal-Wallis test) immunolabeling densities in the midbrain sections was found to be statistically significant. **(e)** The difference between the groups in terms of SNCA (p = 0.058, ANOVA) immunolabeling densities in the midbrain sections was not statistically significant; however, a significant difference was detected between CM and EM groups (● : p = 0.037) with the Tukey HSD test. **(f)** The difference between the groups in terms of phospho(ser129)-SNCA (p < 0.000, ANOVA) immunolabeling densities in the midbrain sections was found to be statistically significant and it was determined that this situation was due to significant differences between CF and EF groups (*: p = 0.002, Tukey HSD), CM and EF groups (▲ : p < 0.000, Tukey HSD) and EF and EM groups (**: p = 0.013, Tukey HSD). **(g)** The difference between the groups in terms of TH (p = 0.033, ANOVA) immunolabeling densities in the midbrain sections was found to be statistically significant and it was determined that this situation was due to significant differences between CM and EM groups (●: p = 0.048, Tukey HSD).

**Fig 8:**
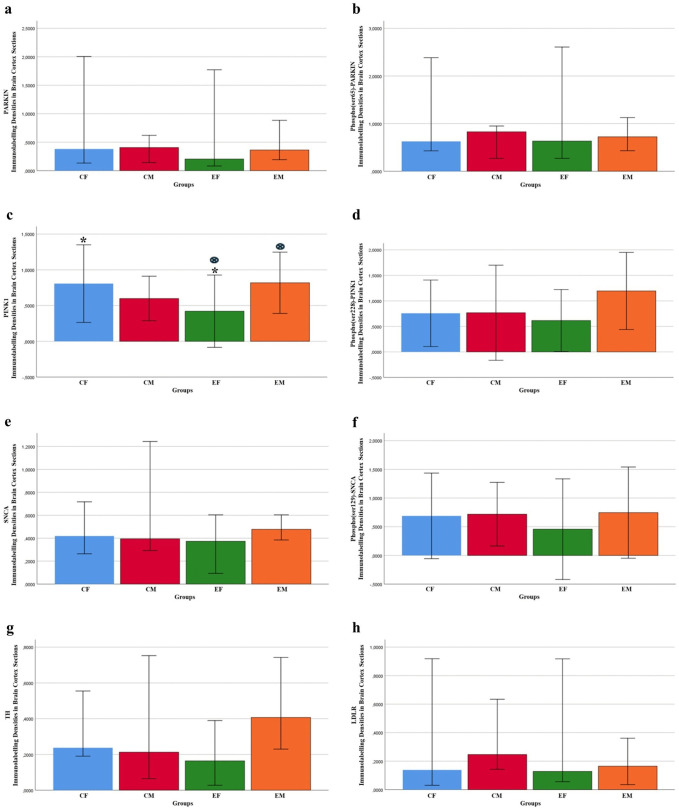
Graphs of multiple comparisons between groups of PARKIN, phospho(ser65)-PARKIN, PINK1, phospho(ser228)-PINK1, SNCA, phospho(ser129)-SNCA, TH and LDLR immunolabeling densities in the brain cortex sections. **(a, b, d-h)** The difference between the groups in terms of PARKIN (p = 0.515, Kruskal-Wallis test), phospho(ser65)-PARKIN (p = 0.885, Kruskal-Wallis test), phospho(ser228)-PINK1 (p = 0.070, ANOVA), SNCA (p = 0.602, Kruskal-Wallis test), phospho(ser129)-SNCA (p = 0.558, ANOVA), TH (p = 0.075, Kruskal-Wallis test) and LDLR (p = 0.562, Kruskal-Wallis test) immunolabeling densities in the brain cortex sections was not statistically significant. **(c)** The difference between the groups in terms of PINK1 (p = 0.023, ANOVA) immunolabeling densities in the brain cortex sections was found to be statistically significant and it was determined that this situation was due to significant differences between CF and EF groups (*: p = 0.042, Tukey HSD) and EF and EM groups (**: p = 0.035, Tukey HSD).

**Fig 9:**
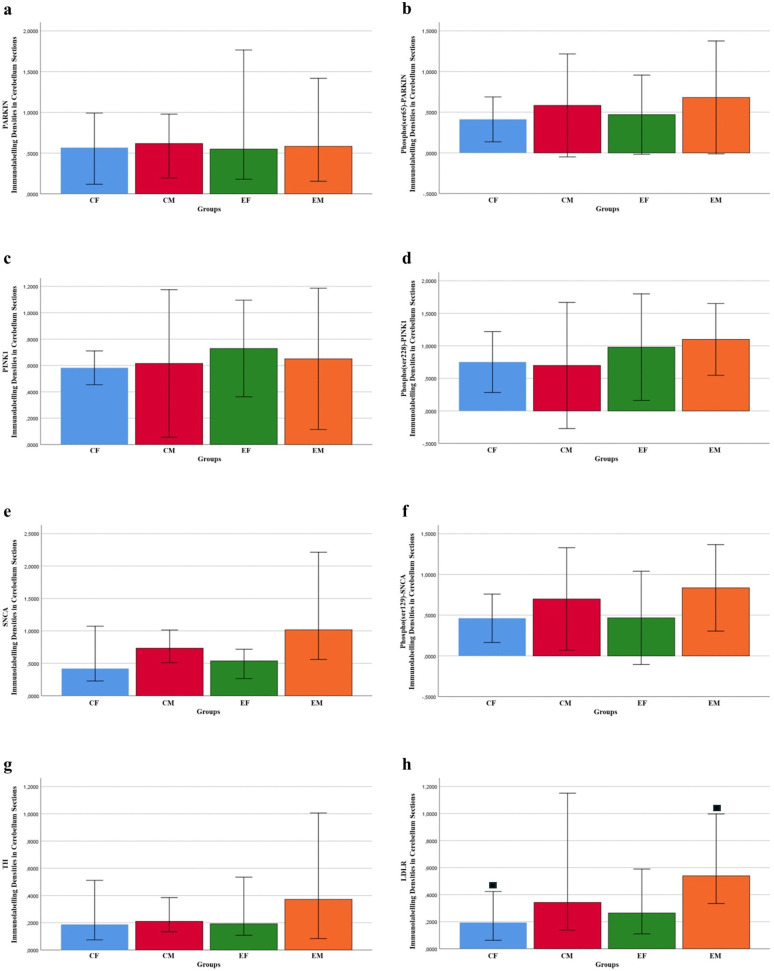
Graphs of multiple comparisons between groups of PARKIN, phospho(ser65)-PARKIN, PINK1, phospho(ser228)-PINK1, SNCA, phospho(ser129)-SNCA, TH and LDLR immunolabeling densities in the cerebellum sections. **(a-g)** The difference between the groups in terms of PARKIN (p = 0.992, Kruskal-Wallis test), phospho(ser65)-PARKIN (p = 0.349, ANOVA), PINK1 (p = 0.675, ANOVA), phospho(ser228)-PINK1 (p = 0.224, ANOVA), SNCA (p = 0.072, Kruskal-Wallis test), phospho(ser129)-SNCA (p = 0.057, ANOVA) and TH (p = 0.811, Kruskal-Wallis test) immunolabeling densities in the cerebellum sections was not statistically significant. **(h)** The difference between the groups in terms of LDLR (p = 0.031, Kruskal-Wallis test) immunolabeling densities in the cerebellum sections was found to be statistically significant and it was determined that this situation was due to significant differences between CF and EM groups (■ : p = 0.050, Kruskal-Wallis test).

There was no statistically significant difference between the groups in terms of PARKIN immunolabeling in the midbrain, brain cortex and cerebellum sections (p = 0.945, p = 0.515 and p = 0.992, respectively). While there was a statistically significant difference between the groups in terms of PINK1 immunolabeling in the midbrain and brain cortex sections (p = 0.027 and p = 0.023, respectively), there was no statistically significant difference between the groups in terms of PINK1 immunolabeling in the cerebellum sections (p = 0.675). When multiple comparisons were made between groups, no statistically significant difference was found between the groups in terms of SNCA immunolabeling in the midbrain sections (p = 0.058); however, a significant difference was detected between the CM group and the EM group (p = 0.037) using the Tukey’s HSD post hoc test. There was no statistically significant difference between the groups in terms of SNCA immunolabeling in the brain cortex and cerebellum sections (p = 0.602 and p = 0.072, respectively). While there was no statistically significant difference between the groups in terms of LDLR immunolabeling in the midbrain and brain cortex sections (p = 0.582 and p = 0.562, respectively), there was a statistically significant difference between the groups in terms of LDLR immunolabeling in the cerebellum sections (p = 0.031). There was no statistically significant difference between the groups in terms of phospho(ser65)-PARKIN immunolabeling in the midbrain, brain cortex and cerebellum sections (p = 0.188, p = 0.885 and p = 0.349, respectively). While there was a statistically significant difference between the groups in terms of phospho(ser228)-PINK1 immunolabeling in the midbrain sections (p = 0.031), there was no statistically significant difference between the groups in terms of phospho(ser228)-PINK1 immunolabeling in the brain cortex and cerebellum sections (p = 0.070 and p = 0.224, respectively). There was a statistically significant difference between the groups in terms of phospho(ser129)-SNCA immunolabeling in the midbrain sections (p < 0.000), but there was no statistically significant difference between the groups in terms of phospho(ser129)-SNCA immunolabeling in the brain cortex and cerebellum sections (p = 0.558 and p = 0.057, respectively). There was a statistically significant difference between the groups in terms of TH immunolabeling in the midbrain sections (p = 0.033), but there was no statistically significant difference between the groups in terms of TH immunolabeling in the brain cortex and cerebellum sections (p = 0.075 and p = 0.811, respectively). The p values of pairwise and multiple comparisons of PARKIN, PINK1, SNCA, LDLR, phospho(ser65)-PARKIN, phospho(ser228)-PINK1, phospho(ser129)-SNCA and TH immunolabeling in the midbrain, brain cortex and cerebellum sections are given in S9 Table. Photographs showing PARKIN and phospho(ser65)-PARKIN immunolabeling in the midbrain, brain cortex and cerebellum sections of the groups are given in Fig 3. Photographs showing PINK1 and phospho(ser228)-PINK1 immunolabeling in the midbrain, brain cortex and cerebellum sections of the groups are given in Fig 4. Photographs showing SNCA and phospho(ser129)-SNCA immunolabeling in the midbrain, brain cortex and cerebellum sections of the groups are given in Fig 5. Photographs showing LDLR and TH immunolabeling in the midbrain, brain cortex and cerebellum sections of the groups are given in Fig 6. Graphs for the comparison of PARKIN, PINK1, SNCA, LDLR, phospho(ser65)-PARKIN, phospho(ser228)-PINK1, phospho(ser129)-SNCA and TH immunolabeling in the midbrain, brain cortex and cerebellum sections according to the groups are given in Figs 7–9.

### Biochemical analysis results

Statistically significant differences were found between the groups in terms of CHOL, LDL, HDL and GLU levels in the serum samples (p < 0.000, p < 0.000, p = 0.001 and p = 0.006, respectively). The p values of pairwise and multiple comparisons of CHOL, LDL, HDL and GLU levels in the serum are given in S10 Table. The graphs related to the comparison of CHOL, LDL, HDL and GLU levels measured in the serum samples according to the groups are given in Fig 1.

### ELISA results

There was no statistically significant difference between the groups in terms of Apo B levels in the serum, brain and cerebellum tissues (p = 0.271, p = 0.427 and p = 0.790, respectively). The p values of pairwise and multiple comparisons of Apo B levels in the serum, brain and cerebellum tissues are given in S10 Table. The graphs related to the comparison of Apo B levels determined in the serum, brain and cerebellum tissues according to the groups are given in Fig 1.

### RT qPCR results

**Fig 10:**
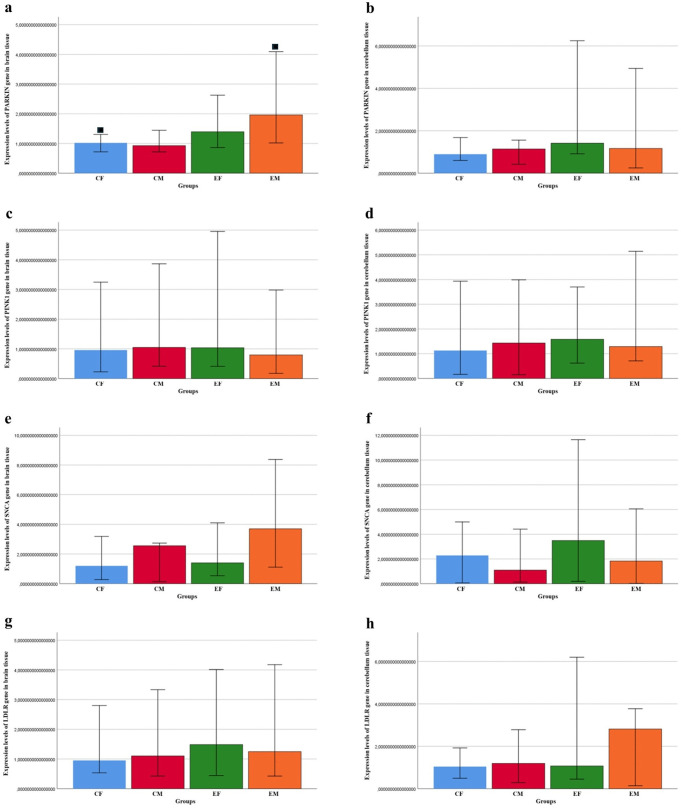
Graphs of multiple comparisons between groups of PARKIN, PINK1, SNCA and LDLR genes expression levels in the brain and cerebellum tissues. (a) A statistically significant difference was found in terms of PARKIN gene expression in the brain tissues (p=0.030, Kruskal-Wallis test). It was determined that this situation was due to the significant difference between CF and EM groups (■ : p=0.050, Kruskal-Wallis test). (b) There was no statistically significant difference between the groups in terms of the expression of PARKIN gene in the cerebellum tissue (p = 0.444, Kruskal-Wallis test). (c, d) There was no statistically significant difference between the groups in terms of the expression of PINK1 gene in the brain (p = 0.975, Kruskal-Wallis test) and cerebellum (p=0.960, Kruskal-Wallis test) tissues. (e, f) There was no statistically significant difference between the groups in terms of the expression of SNCA gene in the brain (p = 0.264, Kruskal-Wallis test) and cerebellum (p = 0.677, Kruskal-Wallis test) tissues. (g, h) There was no statistically significant difference between the groups in terms of the expression of LDLR gene in the brain (p = 0.737, Kruskal-Wallis test) and cerebellum (p = 0.428, Kruskal-Wallis test) tissues.

There was a statistically significant difference between the groups in terms of PARKIN gene expression in the brain tissues (p = 0.030), but no significant difference was found between the groups in terms of PARKIN gene expression in the cerebellum tissues (p = 0.444). There was no statistically significant difference between the groups in terms of the expression of PINK1, SNCA and LDLR genes in the brain (p = 0.975, p = 0.264 and p = 0.737, respectively) and cerebellum tissues (p = 0.960, p = 0.677 and p = 0.428, respectively). The p values of pairwise and multiple comparisons of PARKIN, PINK1, SNCA and LDLR expressions in the brain and cerebellum tissues are given in S10 Table. The graphs of PARKIN, PINK1, SNCA and LDLR gene expression levels in the brain and cerebellum tissues are given in Fig 10.

### Correlation analysis results

A strong positive significant correlation was found between CHOL levels in the serum and phospho(ser228)-PINK1 and TH immunolabeling in the midbrain sections (r = 0.581, p = 0.004 and r = 0.501, p = 0.015, respectively). A strong positive correlation was detected between LDL levels in the serum and phospho(ser228)-PINK1immunolabeling in the midbrain sections (r = 0.596, p = 0.003). There was a strong positive correlation between HDL levels in the serum and SNCA, phospho(ser129)-SNCA, phospho(ser228)-PINK1 and TH immunolabeling in the midbrain sections (r = 0.514, p = 0.012; r = 0.604, p = 0.002; r = 0.613; p = 0.002 and r = 0.534, p = 0.009 respectively). No significant correlations were observed between GLU levels in the serum and immunohistochemical markers in the midbrain and brain cortex sections or between CHOL, LDL and HDL levels in the serum and immunohistochemical markers in the brain cortex and cerebellum sections (all p > 0.05). No significant correlation was found between the expression levels of PARKIN, PINK1, SNCA and LDLR genes in the brain and cerebellum tissues and CHOL, LDL and GLU levels in the serum (all p > 0.05). There was a strong positive correlation between phospho(ser65)-PARKIN immunolabeling in the brain cortex sections and Apo B levels in the brain tissues (r = 0.534, p = 0.009). No significant correlations were observed between Apo B levels in the serum, brain and cerebellum tissues and immunohistochemical markers in the cerebellum sections and gene expression levels in the brain and cerebellum tissues (all p > 0.05). No significant correlation was detected between CHOL, LDL, HDL and GLU levels in the serum and Apo B levels in the serum and brain tissues (all p > 0.05). No significant correlation was detected between CHOL, LDL and HDL levels in the serum and Apo B levels in the cerebellum tissues (all p > 0.05). There was a strong negative correlation between GLU levels in the serum and Apo B levels in the cerebellum tissues (r = 0.515, p = 0.012). Strong positive correlations were observed among CHOL, LDL, and HDL levels in the serum (all + 0.5 < r<+1.0, p < 0.000). Correlation coefficients and p values of the correlation analysis results are given in S11, S12 and S13 Tables.

## Discussion

The present study demonstrated that a high-fat diet containing cholesterol was associated with increased neuronal degeneration in the midbrain and brain cortex sections of the EM group compared with other groups. Furthermore, significant differences were found between the groups in terms of PARKIN expression in the brain tissues and PINK1, phospho(ser228)-PINK1, phospho(ser129)-SNCA and TH immunolabeling densities in the midbrain sections. Although no significant correlation was observed between Apo B levels and the expression of the PARKIN, PINK1 and SNCA genes, a limited number of strong associations were identified, particularly between serum lipid parameters and the immunoreactivity of phospho(ser228)-PINK1, SNCA, phospho(ser129)-SNCA and TH in the midbrain sections. Overall, these findings suggest that a high-fat diet containing cholesterol may be associated with molecular and histological changes in the midbrain relevant to neurodegenerative processes.

Compared to other rodents, it has been noted that the lipid metabolism of guinea pigs is more similar to that of humans [64–68]. High-fat diets are commonly used to induce metabolic diseases in guinea pigs [64]. It has been reported that guinea pigs are more selective in their diet than mice and rats and therefore do not tend to develop obesity along with metabolic disorders when fed a high-fat diet [51,64,65]. It is thought that this situation allows the metabolic effects of high-fat diets to be evaluated without the potential contribution of mechanisms associated with obesity [64]. It has been reported that guinea pigs raised in the laboratory and fed *ad libitum* generally become obese with age and restrict their high-fat diet consumption on their own [64]. In our study, when the body weights of the female and male guinea pigs in both the control and experimental groups at the beginning of the experiment were compared with the body weights at the end of the experiment according to time, the body weights of the CF, CM, EF and EM groups increased significantly. When the body weights were compared according to the groups, the body weight of the CM group was significantly higher than the CF, EF and EM groups. Based on the information in the literature, the findings regarding body weights may be attributed to physiological characteristics of guinea pigs.

A high-fat diet is a diet that provides at least 35% of total calories from both unsaturated and saturated fats [69]. It has been reported that short- and long-term high-fat diet feeding is associated with serious chronic neural diseases through changes in dopaminergic neuroplasticity in the brain due to oxidative stress [70–74]. It has been noted that a high-fat diet may provide greater sensitivity to environmental toxins and accelerate the pathogenesis of Parkinson’s disease [75,76]. However, direct evidence linking high-fat diet exposure to Parkinson’s disease in guinea pigs is currently lacking, and most available data are derived from mouse, rat, and human studies, which are relatively limited in number. In a study using adult male C57BL/6 mice, moderate neuronal degeneration in the substantia nigra pars compacta and vascular hyperemia were reported following high-fat diet exposure [77]. In our study, H&E and neutral red staining were performed for histological examination of the midbrain, brain cortex and cerebellum sections. Although these findings show partial consistency with previous rodent studies in terms of diet-related neuronal changes, species-specific differences in lipid metabolism between mice and guinea pigs should be taken into consideration when interpreting the results.

PINK1 and PARKIN signaling play key roles in mitophagy, mitochondrial motility, and mitochondrial size. Defective mitophagy and PINK1/PARKIN signaling are present in neurodegenerative diseases such as Alzheimer’s disease, Parkinson’s disease, and glaucoma [78–83]. Under physiological conditions, PINK1 is continuously imported into mitochondria and degraded; however, under stress conditions such as reduced mitochondrial membrane potential (ΔΨm), PINK1 accumulates on the outer mitochondrial membrane, leading to PARKIN recruitment and activation of mitophagy through phosphorylation and ubiquitination dependent mechanisms [84–88]. Many studies have shown that PINK1 is autophosphorylated in its activated form in mitochondria [89–92]. One study reported that phospho(ser228)-PINK1 phosphorylates mitochondria-bound ubiquitin and cytosolic PARKIN on Ser65, thereby recruiting and activating PARKIN to ubiquitinate outer mitochondrial membrane proteins [93]. However, findings regarding the effects of high-fat diet on this pathway remain inconsistent. While some studies reported decreased PARKIN protein levels without changes in PINK1 expression, others demonstrated increased PINK1 and PARKIN protein levels in the brain under similar conditions [94,95]. In our study, changes in PARKIN mRNA expression were observed in the brain tissues of the EM group compared to the other groups. Furthermore, an increase in PINK1 and phospho(Ser228)-PINK1 immunolabeling was detected in the midbrain sections from the EM group. These findings suggest that a high-fat diet containing cholesterol may be associated with the regulation of mitochondrial quality control mechanisms in male guinea pigs. Furthermore, findings regarding PINK1 immunolabeling in the brain cortex sections and phospho(Ser228)-PINK1 immunolabeling in the cerebellum sections suggest that different brain regions may exhibit varying sensitivities to dietary lipids. However, these findings require further functional validation.

α-Synuclein modulates synaptic transmission and is released when the neuron is stimulated [96,97]. α-Synuclein has a major role in the pathophysiology of Parkinson’s disease [96]. α-Synuclein is an abundant protein in neurons and is minimally phosphorylated at serine 129 [98]. In contrast, in the brains of individuals with Parkinson’s disease, it is estimated that more than 90% of α-synuclein in inclusions may be phosphorylated [99]. Phosphorylation at serine 129 distinguishes normal α-synuclein from abnormal α-synuclein, particularly α-synuclein in proteinaceous inclusions [98]. According to previous studies, a high-fat diet increased α-synuclein mRNA expression in the midbrain of C57BL/6J mice, whereas no change in α-synuclein levels was observed in the ventral tegmental area and substantia nigra of Wistar rats [100,101]. Pathological changes have been reported to occur in the cerebellum following dopaminergic degeneration in Parkinson’s patients and animal models [102]. α-Synuclein is also present in areas not directly associated with Parkinson’s disease, including the cerebellum [103]. Previous studies indicate that Parkinson’s disease is associated with alterations in α-synuclein in the cerebellum, with some human and animal studies reporting decreased expression, while others have reported region-specific increases in immunolabeling [104–107]. In our study, it was observed that a high-fat diet containing cholesterol was associated with changes in SNCA and phospho(Ser129)-SNCA levels in the midbrain sections of male guinea pigs. These findings suggest that dietary lipids may influence synuclein-related processes in brain regions.

The TH enzyme catalyzes the conversion of L-tyrosine to L-DOPA in catecholamine neurons, such as dopaminergic and norepinephrine neurons [20]. Previous studies have reported region and model dependent effects of high-fat diet on TH expression. While high-fat diet exposure has been associated with decreased TH levels and dopaminergic neuron loss in the substantia nigra in several rodent models, some studies have demonstrated region-specific changes, such as reduced TH levels in the ventral tegmental area without alterations in the substantia nigra [20,94,100]. In contrast, increased TH mRNA expression has also been reported in certain brain regions, including the ventral tegmental area and locus coeruleus, under chronic high-fat diet conditions [108]. According to the immunohistochemical findings of the our study, it was found that TH immunolabeling in the midbrain sections of male guinea pigs in the EM group was significantly increased compared to the control groups whereas no significant differences were observed in the brain cortex or cerebellum sections. In contrast, no significant difference in TH immunolabeling was observed in the EF group compared to the control groups. Decreased TH expression is commonly associated with dopaminergic dysfunction and neurodegenerative conditions [109–111]. However, our findings do not support a reduction in TH levels; on the contrary, they indicate an increase in the midbrain sections of the EM group. This suggests that a high-fat diet may affect dopaminergic markers in different ways under non-pathological conditions. A possible explanation for this increase may relate to the fatty acid composition of the diet. Short-chain fatty acids have been shown to increase TH expression via a cAMP-dependent mechanism, whereas the role of long-chain saturated and unsaturated fatty acids has not yet been determined [112,113]. Notably, a diet enriched with the saturated free fatty acid palmitic acid has been reported to increase both α-synuclein and TH expression in the brain [114]. Given that the high-fat diet containing cholesterol used in this study contains palm kernel oil, a source of palmitic acid, the observed increase in TH immunolabeling in the EM group may be related to this component. However, the absence of a similar effect in the EF group suggests a potential sex-dependent response. But this interpretation should be made cautiously. Collectively, these results suggest that high-fat diet induced changes in TH expression may reflect an adaptive or regulatory response rather than a pathological increase, highlighting the need for further studies to clarify the underlying mechanisms.

LDLR is an important receptor for Apo E in the central nervous system [115]. LDLR, which induces Apo E and Aβ uptake, is expressed in astrocytes and has been reported to prevent Aβ accumulation by reducing Apo E levels in mice overexpressing LDLR [116,117]. Aβ, known to cause Alzheimer’s disease, accumulates in the brain as plaques and is assumed to be regulated by receptors for Apo E [118–120]. In addition, experimental studies have shown that LDLR deficiency may contribute to cognitive impairment and increased oxidative stress in transgenic models [121–124]. To our knowledge, there are no studies in guinea pigs evaluating LDLR levels in relation to high-fat diets containing cholesterol or Parkinson’s disease–related conditions. In our study, we found that the density of LDLR immunolabelling in the cerebellum sections of male guinea pigs in the EM group, which were fed a high-fat diet containing cholesterol, was increased. The relatively limited effect observed in the cerebellum may suggest a potential compensatory role of LDLR-related mechanisms.

The effects of dietary fatty acids on cholesterol and lipoprotein metabolism have been well documented in guinea pigs, particularly in relation to LDL levels and Apo B secretion [43,125]. Studies have shown that diets enriched with different fat sources, such as palm kernel oil, lard, or corn oil, can significantly alter lipoprotein composition and metabolism [43]. However, the distribution of lipids across lipoprotein fractions differs significantly between animal models and humans [126]. In our study, Apo B levels in the serum, brain and cerebellum tissues were not significantly different among the groups. In contrast, serum analyses showed significant increases in CHOL, LDL and HDL levels in the experimental groups, indicating diet-induced dyslipidemia. This observation suggests that Apo B levels in the serum and tissues may be relatively resistant to dietary modulation, or that changes in serum lipid profiles are not directly mirrored in tissues. The highest GLU levels were observed in the CM group. This finding may be related to physiological variability in laboratory-reared guinea pigs and potential differences in body weight under standard chow diet conditions.

Different blood lipid fractions have been identified as risk factors for Parkinson’s disease [32]. Previous studies have reported associations between blood lipid fractions and Parkinson’s disease risk, although findings remain inconsistent regarding the role of Apo B and cholesterol-related parameters [30,127–131]. In our study, although no significant correlations were observed between Apo B levels and the expression of PARKIN, PINK1, and SNCA genes, the expression levels of these genes were altered in response to the cholesterol-containing high-fat diet. A limited number of strong and statistically significant correlations were nevertheless identified. A significant positive correlation was observed between phospho(ser65)-PARKIN immunolabeling in the brain cortex sections and Apo B levels in the brain tissues. Serum CHOL, LDL and HDL levels showed strong positive correlations with phospho(ser228)-PINK1, SNCA, phospho(ser129)-SNCA and TH immunolabeling in the midbrain sections. Serum CHOL, LDL, and HDL levels were also positively correlated with each other. In addition, serum GLU levels showed a strong negative correlation with Apo B levels in the cerebellum tissues. Alltogether, these findings suggest that the observed correlations are limited and may reflect potential regional trends, particularly in the midbrain.

The present study has several limitations that should be considered when interpreting the findings. First, the relatively small sample size, distributed across four groups, may have reduced statistical power. Second, the inclusion of multiple molecular, biochemical, and histological endpoints may increase the likelihood of exploratory or false-positive findings. Third, the absence of functional assays limits mechanistic interpretation of the results. Fourth, the failure to measure individual food intake may lead to variability in dietary exposure. In addition, the study did not employ a Parkinson’s disease model; therefore, the findings should not be interpreted as direct evidence of Parkinsonian pathology but rather as diet-induced modulation of neurobiological pathways relevant to neurodegeneration. Overall, these results should be interpreted as exploratory and hypothesis-generating.

Future studies should further investigate these findings using larger sample sizes and functional assays, as well as established Parkinson’s disease models, to better elucidate the mechanistic relevance of the observed diet-induced changes. Sex- and region-related effects should be examined in more detail using comprehensive experimental designs.

## Conclusion

In conclusion, the present study suggests that a high-fat diet containing cholesterol is associated with alterations in Parkinson’s disease–related genes and neurobiological markers in male guinea pigs, particularly involving PARKIN expression. These findings indicate that dietary lipid exposure may modulate molecular pathways associated with neurodegeneration. However, as no Parkinson’s disease model was used, the results should not be interpreted as evidence of Parkinson’s disease pathology, but rather as changes in PD-related molecular targets. Further studies incorporating larger sample sizes and functional disease models are required to validate these observations.

## Supporting information

S1 Table Dilution rates of primary antibodies. (PDF)

S2 Table Device protocol for reverse transcription process. (PDF)

S3 Table Primer sequences used in the study. (PDF)

S4 Table Device protocol used for RT-qPCR. (PDF)

S5 Table Mauchly’s sphericity test within the scope of repeated measures ANOVA for body weights. (PDF)

S6 Table Pairwise comparisons of body weights according to weeks. (PDF)

S7 Table Pairwise comparisons of body weights according to groups. (PDF)

S8 Table Multiple comparisons of body weights according to groups. (PDF)

S9 Table The p values of pairwise and multiple comparisons of PARKIN, PINK1, SNCA, LDLR, phospho(ser65)-PARKIN, phospho(ser228)-PINK1, phospho(ser129)-SNCA and TH immunolabeling in the midbrain, brain cortex and cerebellum sections. (PDF)

S10 Table P values of statistical comparisons based on biochemical analysis, ELISA and real time qPCR results. (PDF)

S11 Table Correlation analysis of PARKIN, PINK1, SNCA, LDLR, phospho(ser65)-PARKIN, phospho(ser228)-PINK1, phospho(ser129)-SNCA and TH immunolabeling in the midbrain, brain cortex and cerebellum sections, expression levels of PARKIN, PINK1, SNCA and LDLR genes in the brain and cerebellum tissues and CHOL, LDL, HDL and GLU levels in the serum. (PDF)

S12 Table Correlation analysis of PARKIN, PINK1, SNCA, LDLR, phospho(ser65)-PARKIN, phospho(ser228)-PINK1, phospho(ser129)-SNCA and TH immunolabeling in the midbrain, brain cortex and cerebellum sections, expression levels of PARKIN, PINK1, SNCA and LDLR genes in the brain and cerebellum tissues and Apo B levels in the serum, brain and cerebellum tissues. (PDF)

S13. Table Correlation analysis of CHOL, LDL, HDL and GLU levels in the serum and Apo B levels in the serum, brain and cerebellum tissues. (PDF)
