# Supplementary material for: Investigation of correlation between cholesterol intake, apolipoprotein B and Parkinson’s disease related genes in guinea pigs feeding a high-fat diet containing cholesterol
Source: PLoS One. 2026 Jun 25;21(6):e0352642. doi: 10.1371/journal.pone.0352642 (PMC13298788; doi:10.1371/journal.pone.0352642)
Supplement: S1 Table — (PDF) [file pone.0352642.s001.pdf]

**S1 Table. Dilution rates of primary antibodies**		
**Primary Antibody**	**Dilution Rate**	
	**Midbrain, Brain cortex**	**Cerebellum**
PARKIN	1/1000	1/1000
PINK1	1/50	1/300
SNCA	1/100	1/200
LDLR	1/200	1/200
Phospho(ser(65)-PARKIN	1/300	1/300
Phospho(ser228)-PINK1	1/50	1/50
Phospho(129)-SNCA	1/50	1/50
Tyrosine Hydroxylase	1/200	1/200
