# Supplementary material for: Investigation of correlation between cholesterol intake, apolipoprotein B and Parkinson’s disease related genes in guinea pigs feeding a high-fat diet containing cholesterol
Source: PLoS One. 2026 Jun 25;21(6):e0352642. doi: 10.1371/journal.pone.0352642 (PMC13298788; doi:10.1371/journal.pone.0352642)
Supplement: S2 Table — (PDF) [file pone.0352642.s002.pdf]

S2 Table. Device protocol for reverse transcription process		
**Temperature/Degree**	**Time**	**Process**
25 ℃	10 min	primer binding
42 ℃	15 min	reverse transcription process
85 ℃	5 min	enzyme inactivation
