# Supplementary material for: Investigation of correlation between cholesterol intake, apolipoprotein B and Parkinson’s disease related genes in guinea pigs feeding a high-fat diet containing cholesterol
Source: PLoS One. 2026 Jun 25;21(6):e0352642. doi: 10.1371/journal.pone.0352642 (PMC13298788; doi:10.1371/journal.pone.0352642)
Supplement: S3 Table — (PDF) [file pone.0352642.s003.pdf]

**S3 Table. Primer sequences used in the study**		
**Gene Name**	**Oligo Name (Primary)**	**Primer Sequence**
PARKIN	PARKIN_cpo_F	5’ CCTAATAACAACCAACAG 3’
PARKIN	PARKIN_cpo_R	5’ AAACAGTCTAAGCAAATC 3’
LDLR	LDLR_cpo_F	5’ CAGGCTTGGATGTTCATT 3’
LDLR	LDLR_cpo_R	5’ TACTTTCACACCAGTTCAC 3’
PINK1	PINK1_cpo_F	5’ GTATGTGGATCGAGGTGG 3’
PINK1	PINK1_cpo_R	5’ TTGCTGTAGTCAATCACTG 3’
SNCA	SNCA_cpo_F	5’^GGTGTTCTCTATGTAGGC 3’
SNCA	SNCA_cpo_R	5’ CATTTGTTACTTGTTCTTTGG 3’
RPS16	RPS16-cpo-F	5’ CTCAGATTTACGCTATCC 3’
RPS16	RPS16-cpo-R	5’ AATATCTTTGATCTCTTTCTTG 3’
