# Supplementary material for: Investigation of correlation between cholesterol intake, apolipoprotein B and Parkinson’s disease related genes in guinea pigs feeding a high-fat diet containing cholesterol
Source: PLoS One. 2026 Jun 25;21(6):e0352642. doi: 10.1371/journal.pone.0352642 (PMC13298788; doi:10.1371/journal.pone.0352642)
Supplement: S4 Table — (PDF) [file pone.0352642.s004.pdf]

S4 Table. Device protocol used for RT-qPCR

PCR Stage	Process	Temperature/Degree	Time	Number of repetitions
Initial Denaturation	Denaturation	95 ℃	5 min	1
Amplification	Denaturation	95 ℃	5 sec	40
	Binding	60 ℃	30 sec	
Final	Cooling	40 ℃	30 sec	1
