# Supplementary material for: Investigation of correlation between cholesterol intake, apolipoprotein B and Parkinson’s disease related genes in guinea pigs feeding a high-fat diet containing cholesterol
Source: PLoS One. 2026 Jun 25;21(6):e0352642. doi: 10.1371/journal.pone.0352642 (PMC13298788; doi:10.1371/journal.pone.0352642)
Supplement: S5 Table — (PDF) [file pone.0352642.s005.pdf]

S5 Table. Mauchly's sphericity test within the scope of repeated measures ANOVA for body weights		
**Mauchly's Sphericity Test**		
**Factor**	**Measurement Name**	**P Value**
Time	Weight	**0,000**

A value of $p \leq 0.05$ is considered statistically significant and highlighted in bold characters.
