# Supplementary material for: Investigation of correlation between cholesterol intake, apolipoprotein B and Parkinson’s disease related genes in guinea pigs feeding a high-fat diet containing cholesterol
Source: PLoS One. 2026 Jun 25;21(6):e0352642. doi: 10.1371/journal.pone.0352642 (PMC13298788; doi:10.1371/journal.pone.0352642)
Supplement: S6 Table — (PDF) [file pone.0352642.s006.pdf]

S6 Table. Pairwise comparisons of body weights according to weeks								
**Week**	**Week**	**P value***	**Week**	**Week**	**P value***	**Week**	**Week**	**P value***
SOE	1	**0,000**	5	SOE	**0,000**	9	SOE	**0,000**
	2	**0,000**		1	**0,000**		1	**0,000**
	3	**0,000**		2	**0,000**		2	**0,000**
	4	**0,000**		3	**0,000**		3	**0,000**
	5	**0,000**		4	**0,000**		4	**0,000**
	6	**0,000**		6	1,000		5	**0,003**
	7	**0,000**		7	0,422		6	**0,000**
	8	**0,000**		8	**0,000**		7	**0,004**
	9	**0,000**		9	**0,003**		8	1,000
	10	**0,000**		10	**0,000**		10	**0,000**
	11	**0,000**		11	**0,002**		11	**0,022**
	12	**0,000**		12	**0,003**		12	**0,042**
1	SOE	**0,000**	6	SOE	**0,000**	10	SOE	**0,000**
	2	**0,001**		1	**0,000**		1	**0,000**
	3	**0,001**		2	**0,000**		2	**0,000**
	4	**0,000**		3	**0,000**		3	**0,000**
	5	**0,000**		4	**0,000**		4	**0,000**
	6	**0,000**		5	1,000		5	**0,000**
	7	**0,000**		7	**0,000**		6	**0,000**
	8	**0,000**		8	**0,000**		7	**0,000**
	9	**0,000**		9	**0,000**		8	**0,002**
	10	**0,000**		10	**0,000**		9	**0,000**
	11	**0,000**		11	**0,000**		10	1,000
	12	**0,000**		12	**0,001**		11	1,000
2	SOE	**0,000**	7	SOE	**0,000**	11	SOE	**0,000**
	1	**0,001**		1	**0,000**		1	**0,000**
	3	0,136		2	**0,000**		2	**0,000**
	4	**0,000**		3	**0,000**		3	**0,000**
	5	**0,000**		4	**0,000**		4	**0,000**
	6	**0,000**		5	0,422		5	**0,002**
	7	**0,000**		6	**0,000**		6	**0,000**
	8	**0,000**		8	**0,009**		7	**0,003**
	9	**0,000**		9	**0,004**		8	**0,029**
	10	**0,000**		10	**0,000**		9	**0,022**
	11	**0,000**		11	**0,003**		10	1,000
	12	**0,000**		12	**0,008**		12	1,000
3	SOE	**0,000**	8	SOE	**0,000**	12	SOE	**0,000**
	1	**0,001**		1	**0,000**		1	**0,000**
	2	0,136		2	**0,000**		2	**0,000**
	4	**0,000**		3	**0,000**		3	**0,000**
	5	**0,000**		4	**0,000**		4	**0,000**
	6	**0,000**		5	**0,000**		5	**0,003**
	7	**0,000**		6	**0,000**		6	**0,001**
	8	**0,000**		7	**0,009**		7	**0,008**
	9	**0,000**		9	1,000		8	**0,043**
	10	**0,000**		10	**0,002**		9	**0,042**
	11	**0,000**		11	**0,029**		10	1,000
	12	**0,000**		12	**0,043**		11	1,000
4	SOE	**0,000**						
	1	**0,000**						
	2	**0,000**						
	3	**0,000**						
	5	**0,000**						
	6	**0,000**						
	7	**0,000**						
	8	**0,000**						
	9	**0,000**						
	10	**0,000**						
	11	**0,000**						
	12	**0,000**						

A value of $p \leq 0.05$ was considered statistically significant. *Bonferroni correction was performed. SOE: start of experiment
