# Supplementary material for: Investigation of correlation between cholesterol intake, apolipoprotein B and Parkinson’s disease related genes in guinea pigs feeding a high-fat diet containing cholesterol
Source: PLoS One. 2026 Jun 25;21(6):e0352642. doi: 10.1371/journal.pone.0352642 (PMC13298788; doi:10.1371/journal.pone.0352642)
Supplement: S7 Table — (PDF) [file pone.0352642.s007.pdf]

**S7 Table. Pairwise comparisons of body weights according to groups**		
**Groups**	**Groups**	**P value***
CF	CM	**0,000**
	EF	1,000
	EM	1,000
CM	CF	**0,000**
	EF	**0,000**
	EM	**0,002**
EF	CF	1,000
	CM	**0,000**
	EM	0,284
EM	CF	1,000
	CM	**0,002**
	EF	0,284

A value of p≤0.05 was considered statistically significant.

*Bonferroni correction was performed.
