# Supplementary material for: Investigation of correlation between cholesterol intake, apolipoprotein B and Parkinson’s disease related genes in guinea pigs feeding a high-fat diet containing cholesterol
Source: PLoS One. 2026 Jun 25;21(6):e0352642. doi: 10.1371/journal.pone.0352642 (PMC13298788; doi:10.1371/journal.pone.0352642)
Supplement: S8 Table — (PDF) [file pone.0352642.s008.pdf]

**S8 Table. Multiple comparisons of body weights according to groups**			
**Post hoc test**	**Groups**	**Groups**	**P value**
Tukey HSD	CF	CM	**0,000**
		EF	0,686
		EM	0,747
Tukey HSD	CM	CF	**0,000**
		EF	**0,000**
		EM	**0,002**
Tukey HSD	EF	CF	0,686
		CM	**0,000**
		EM	0,182
Tukey HSD	EM	CF	0,747
		CM	**0,002**
		EF	0,182

A value of $p \leq 0.05$ was considered statistically significant.
