# Supplementary material for: Investigation of correlation between cholesterol intake, apolipoprotein B and Parkinson’s disease related genes in guinea pigs feeding a high-fat diet containing cholesterol
Source: PLoS One. 2026 Jun 25;21(6):e0352642. doi: 10.1371/journal.pone.0352642 (PMC13298788; doi:10.1371/journal.pone.0352642)
Supplement: S9 Table — (PDF) [file pone.0352642.s009.pdf]

S9 Table. The p values of pairwise and multiple comparisons of PARKIN, PINK1, SNCA, LDLR, phospho(ser65)-PARKIN, phospho(ser228)-PINK1, phospho(ser129)-SNCA and TH immunolabelling in the midbrain, brain cortex and cerebellum sections

	PARKIN (P value)				PINK1 (P value)		
	**Midbrain**	**Brain cortex**	**Cerebellum**		**Midbrain**	**Brain cortex**	**Cerebellum**
**CF / CM**	0,944$^{\alpha}$	0,931$^{\beta}$	0,931$^{\beta}$	**CF / CM**	0,683$^{\alpha}$	0,165$^{\alpha}$	0,806$^{\alpha}$
**CF / EF**	0,506$^{\alpha}$	0,180$^{\beta}$	0,937$^{\beta}$	**CF / EF**	0,716$^{\alpha}$	**0,029$^{\alpha}$**	0,112$^{\alpha}$
**CF / EM**	0,817$^{\alpha}$	0,818$^{\beta}$	0,818$^{\beta}$	**CF / EM**	**0,031$^{\alpha}$**	0,934$^{\alpha}$	0,567$^{\alpha}$
**CM / EF**	0,673$^{\alpha}$	0,429$^{\beta}$	0,931$^{\beta}$	**CM / EF**	0,441$^{\alpha}$	0,205$^{\alpha}$	0,439$^{\alpha}$
**CM / EM**	0,908$^{\alpha}$	0,931$^{\beta}$	0,931$^{\beta}$	**CM / EM**	0,056$^{\alpha}$	0,089$^{\alpha}$	0,836$^{\alpha}$
**EF / EM**	0,743$^{\alpha}$	0,310$^{\beta}$	1,000$^{\beta}$	**EF / EM**	**0,018$^{\alpha}$**	**0,015$^{\alpha}$**	0,568$^{\alpha}$
**CG / EG**	0,608$^{\alpha}$	0,379$^{\beta}$	0,976$^{\beta}$	**CG / EG**	0,259$^{\alpha}$	0,432$^{\alpha}$	0,292$^{\alpha}$
**CF/CM/EF/EM**	0,945$^{\gamma}$	0,515$^{\delta}$	0,992$^{\delta}$	**CF/CM/EF/EM**	**0,027$^{\gamma}$**	**0,023$^{\gamma}$**	0,675$^{\gamma}$
	**Phospho(ser65)-PARKIN (P value)**				**Phospho(ser228)-PINK1 (P value)**		
	**Midbrain**	**Brain cortex**	**Cerebellum**		**Midbrain**	**Brain cortex**	**Cerebellum**
**CF / CM**	0,537$^{\beta}$	0,429$^{\beta}$	0,258$^{\alpha}$	**CF / CM**	0,429$^{\beta}$	0,965$^{\alpha}$	0,817$^{\alpha}$
**CF / EF**	0,937$^{\beta}$	0,937$^{\beta}$	0,625$^{\alpha}$	**CF / EF**	0,485$^{\beta}$	0,454$^{\alpha}$	0,269$^{\alpha}$
**CF / EM**	0,240$^{\beta}$	0,699$^{\beta}$	0,124$^{\alpha}$	**CF / EM**	**0,004$^{\beta}$**	0,057$^{\alpha}$	**0,040$^{\alpha}$**
**CM / EF**	0,247$^{\beta}$	0,931$^{\beta}$	0,517$^{\alpha}$	**CM / EF**	0,177$^{\beta}$	0,529$^{\alpha}$	0,322$^{\alpha}$
**CM / EM**	0,052$^{\beta}$	0,931$^{\beta}$	0,640$^{\alpha}$	**CM / EM**	**0,017$^{\beta}$**	0,127$^{\alpha}$	0,117$^{\alpha}$
**EF / EM**	0,240$^{\beta}$	0,589$^{\beta}$	0,250$^{\alpha}$	**EF / EM**	0,394$^{\beta}$	**0,015$^{\alpha}$**	0,566$^{\alpha}$
**CG / EG**	0,134$^{\beta}$	0,928$^{\beta}$	0,466$^{\alpha}$	**CG / EG**	**0,007$^{\beta}$**	0,415$^{\alpha}$	**0,041$^{\alpha}$**
**CF/CM/EF/EM**	0,188$^{\delta}$	0,885$^{\delta}$	0,349$^{\gamma}$	**CF/CM/EF/EM**	**0,031$^{\delta}$**	0,070$^{\gamma}$	0,224$^{\gamma}$
	**SNCA (P value)**				**TH (P value)**		
	**Midbrain**	**Brain cortex**	**Cerebellum**		**Midbrain**	**Brain cortex**	**Cerebellum**
**CF / CM**	0,158$^{\alpha}$	1,000$^{\beta}$	0,247$^{\beta}$	**CF / CM**	0,760$^{\alpha}$	0,931$^{\beta}$	0,792$^{\beta}$
**CF / EF**	0,894$^{\alpha}$	0,485$^{\beta}$	0,937$^{\beta}$	**CF / EF**	0,231$^{\alpha}$	0,180$^{\beta}$	0,699$^{\beta}$
**CF / EM**	0,060$^{\alpha}$	0,589$^{\beta}$	0,065$^{\beta}$	**CF / EM**	**0,045$^{\alpha}$**	0,132$^{\beta}$	0,485$^{\beta}$
**CM / EF**	0,325$^{\alpha}$	0,537$^{\beta}$	0,126$^{\beta}$	**CM / EF**	0,063$^{\alpha}$	0,537$^{\beta}$	0,662$^{\beta}$
**CM / EM**	**0,008$^{\alpha}$**	0,537$^{\beta}$	0,329$^{\beta}$	**CM / EM**	**0,009$^{\alpha}$**	0,177$^{\beta}$	0,662$^{\beta}$
**EF / EM**	0,129$^{\alpha}$	0,240$^{\beta}$	**0,041$^{\beta}$**	**EF / EM**	0,261$^{\alpha}$	**0,015$^{\beta}$**	0,589$^{\beta}$
**CG / EG**	0,084$^{\alpha}$	0,976$^{\beta}$	0,651$^{\beta}$	**CG / EG**	**0,006$^{\alpha}$**	0,786$^{\beta}$	0,651$^{\beta}$
**CF/CM/EF/EM**	0,058$^{\gamma}$	0,602$^{\delta}$	0,072$^{\delta}$	**CF/CM/EF/EM**	**0,033$^{\gamma}$**	0,075$^{\delta}$	0,811$^{\delta}$
	**Phospho(ser129)-SNCA (P value)**				**LDLR (P value)**		
	**Midbrain**	**Brain cortex**	**Cerebellum**		**Midbrain**	**Brain cortex**	**Cerebellum**
**CF / CM**	0,158$^{\alpha}$	0,888$^{\alpha}$	0,178$^{\alpha}$	**CF / CM**	0,155$^{\alpha}$	0,329$^{\beta}$	0,126$^{\beta}$
**CF / EF**	**0,002$^{\alpha}$**	0,349$^{\alpha}$	0,969$^{\alpha}$	**CF / EF**	0,531$^{\alpha}$	0,818$^{\beta}$	0,394$^{\beta}$
**CF / EM**	0,348$^{\alpha}$	0,804$^{\alpha}$	**0,013$^{\alpha}$**	**CF / EM**	0,654$^{\alpha}$	1,000$^{\beta}$	**0,009$^{\beta}$**
**CM / EF**	**0,000$^{\alpha}$**	0,282$^{\alpha}$	0,233$^{\alpha}$	**CM / EF**	0,508$^{\alpha}$	0,329$^{\beta}$	0,429$^{\beta}$
**CM / EM**	**0,036$^{\alpha}$**	0,899$^{\alpha}$	0,454$^{\alpha}$	**CM / EM**	0,301$^{\alpha}$	0,247$^{\beta}$	0,429$^{\beta}$
**EF / EM**	**0,010$^{\alpha}$**	0,261$^{\alpha}$	**0,043$^{\alpha}$**	**EF / EM**	0,813$^{\alpha}$	0,937$^{\beta}$	**0,026$^{\beta}$**
**CG / EG**	**0,001$^{\alpha}$**	0,532$^{\alpha}$	0,514$^{\alpha}$	**CG / EG**	0,918$^{\alpha}$	0,379$^{\beta}$	0,190$^{\beta}$
**CF/CM/EF/EM**	**0,000$^{\gamma}$**	0,558$^{\gamma}$	0,057$^{\gamma}$	**CF/CM/EF/EM**	0,582$^{\gamma}$	0,562$^{\delta}$	**0,031$^{\delta}$**

A value of $p \leq 0.05$ is considered statistically significant and highlighted in bold characters. α: Independent sample t-test, β: Mann-Whitney test, γ: ANOVA, δ: Kruskal-Wallis test
