# Supplementary material for: Investigation of correlation between cholesterol intake, apolipoprotein B and Parkinson’s disease related genes in guinea pigs feeding a high-fat diet containing cholesterol
Source: PLoS One. 2026 Jun 25;21(6):e0352642. doi: 10.1371/journal.pone.0352642 (PMC13298788; doi:10.1371/journal.pone.0352642)
Supplement: S10 Table — (PDF) [file pone.0352642.s010.pdf]

S10 Table. P values of statistical comparisons based on biochemical analysis, ELISA and real time qPCR results

The p values of pairwise and multiple comparisons of CHOL, LDL, HDL and GLU levels in the serum

	**CHOL (P value)**	**LDL (P value)**	**HDL (P value)**	**GLU (P value)**
**CF / CM**	$0,286^{\alpha}$	$0,919^{\alpha}$	$0,792^{\beta}$	**$0,004^{\beta}$**
**CF / EF**	**$0,004^{\alpha}$**	**$0,006^{\alpha}$**	**$0,002^{\beta}$**	$0,240^{\beta}$
**CF / EM**	**$0,008^{\alpha}$**	**$0,001^{\alpha}$**	**$0,002^{\beta}$**	**$0,002^{\beta}$**
**CM / EF**	**$0,005^{\alpha}$**	**$0,007^{\alpha}$**	**$0,004^{\beta}$**	**$0,017^{\beta}$**
**CM / EM**	**$0,010^{\alpha}$**	**$0,000^{\alpha}$**	**$0,017^{\beta}$**	$0,247^{\beta}$
**EF / EM**	$0,806^{\alpha}$	$0,991^{\alpha}$	$0,818^{\beta}$	$0,699^{\beta}$
**CG / EG**	**$0,000^{\alpha}$**	**$0,000^{\alpha}$**	**$0,000^{\beta}$**	$0,740^{\beta}$
**CF/CM/EF/EM**	**$0,000^{\gamma}$**	**$0,000^{\gamma}$**	**$0,001^{\delta}$**	**$0,006^{\delta}$**

The p values of pairwise and multiple comparisons of Apo B levels in the serum, brain and cerebellum

	**Apo B (P value)**		
	**Serum**	**Brain**	**Cerebellum**
**CF / CM**	$0,660^{\alpha}$	$0,627^{\alpha}$	$0,324^{\alpha}$
**CF / EF**	$0,495^{\alpha}$	$0,522^{\alpha}$	$0,644^{\alpha}$
**CF / EM**	**$0,046^{\alpha}$**	$0,149^{\alpha}$	$0,878^{\alpha}$
**CM / EF**	$0,766^{\alpha}$	$0,937^{\alpha}$	$0,670^{\alpha}$
**CM / EM**	$0,124^{\alpha}$	$0,288^{\alpha}$	$0,372^{\alpha}$
**EF / EM**	$0,308^{\alpha}$	$0,218^{\alpha}$	$0,735^{\alpha}$
**CG / EG**	$0,113^{\alpha}$	$0,212^{\alpha}$	$0,886^{\alpha}$
**CF/CM/EF/EM**	$0,271^{\gamma}$	$0,427^{\gamma}$	$0,790^{\gamma}$

The p values of pairwise and multiple comparisons of PARKIN, PINK1, SNCA and LDLR expressions in the brain and cerebellum

	**PARKIN (P value)**			**PINK1 (P value)**	
	**Brain**	**Cerebellum**		**Brain**	**Cerebellum**
**CF / CM**	$1,000^{\beta}$	$0,792^{\beta}$	**CF / CM**	$0,931^{\beta}$	$0,931^{\beta}$
**CF / EF**	$0,132^{\beta}$	$0,132^{\beta}$	**CF / EF**	$1,000^{\beta}$	$0,818^{\beta}$
**CF / EM**	**$0,015^{\beta}$**	$0,818^{\beta}$	**CF / EM**	$0,699^{\beta}$	$0,589^{\beta}$
**CM / EF**	$0,329^{\beta}$	$0,177^{\beta}$	**CM / EF**	$0,855^{\beta}$	$0,931^{\beta}$
**CM / EM**	**$0,017^{\beta}$**	$0,931^{\beta}$	**CM / EM**	$0,792^{\beta}$	$0,931^{\beta}$
**EF / EM**	$0,180^{\beta}$	$0,589^{\beta}$	**EF / EM**	$0,937^{\beta}$	$0,937^{\beta}$
**CG / EG**	**$0,006^{\beta}$**	$0,211^{\beta}$	**CG / EG**	$0,740^{\beta}$	$0,651^{\beta}$
**CF/CM/EF/EM**	**$0,030^{\delta}$**	$0,444^{\delta}$	**CF/CM/EF/EM**	$0,975^{\delta}$	$0,960^{\delta}$

	**SNCA (P value)**			**LDLR (P value)**	
	**Brain**	**Cerebellum**		**Brain**	**Cerebellum**
**CF / CM**	$0,931^{\beta}$	$0,792^{\beta}$	**CF / CM**	$1,000^{\beta}$	$1,000^{\beta}$
**CF / EF**	$0,394^{\beta}$	$0,394^{\beta}$	**CF / EF**	$0,394^{\beta}$	$0,699^{\beta}$
**CF / EM**	$0,132^{\beta}$	$0,937^{\beta}$	**CF / EM**	$0,485^{\beta}$	$0,093^{\beta}$
**CM / EF**	$1,000^{\beta}$	$0,329^{\beta}$	**CM / EF**	$0,429^{\beta}$	$0,792^{\beta}$
**CM / EM**	$0,126^{\beta}$	$0,931^{\beta}$	**CM / EM**	$0,792^{\beta}$	$0,177^{\beta}$
**EF / EM**	$0,240^{\beta}$	$0,485^{\beta}$	**EF / EM**	$1,000^{\beta}$	$0,818^{\beta}$
**CG / EG**	$0,134^{\beta}$	$0,497^{\beta}$	**CG / EG**	$0,288^{\beta}$	$0,151^{\beta}$
**CF/CM/EF/EM**	$0,264^{\delta}$	$0,677^{\delta}$	**CF/CM/EF/EM**	$0,737^{\delta}$	$0,428^{\delta}$

A value of $p \leq 0.05$ is considered statistically significant and highlighted in bold characters. α: Independent sample t-test, β: Mann-Whitney test, γ: ANOVA, δ: Kruskal-Wallis test
