# Supplementary material for: Investigation of correlation between cholesterol intake, apolipoprotein B and Parkinson’s disease related genes in guinea pigs feeding a high-fat diet containing cholesterol
Source: PLoS One. 2026 Jun 25;21(6):e0352642. doi: 10.1371/journal.pone.0352642 (PMC13298788; doi:10.1371/journal.pone.0352642)
Supplement: S11 Table — (PDF) [file pone.0352642.s011.pdf]

S11 Table. Correlation analysis of PARKIN, PINK1, SNCA, LDLR, phospho(ser65)-PARKIN, phospho(ser228)-PINK1, phospho(ser129)-SNCA and TH immunolabelling in the midbrain, brain cortex and cerebellum sections, expression levels of PARKIN, PINK1, SNCA and LDLR genes in the brain and cerebellum tissues and CHOL, LDL, HDL and GLU levels in the serum

n=23			Biochemical Analysis			
			Serum			
			CHOL	LDL	HDL	GLU
			R veya R_s / P	R veya R_s / P	R veya R_s / P	R veya R_s / P
Immunohistochemical Analysis	Midbrain	PARKIN	0,217^{ε} / 0,319	0,117^{ε} / 0,594	0,150^{ς} / 0,495	-0,251^{ς} / 0,247
		PINK1	0,288^{ε} / 0,183	0,408^{ε} / 0,053	0,336^{ς} / 0,118	0,325^{ς} / 0,131
		SNCA	**0,459^{ε} / 0,028**	**0,493^{ε} / 0,017**	**0,514^{ς} / 0,012**	0,141^{ς} / 0,521
		LDLR	-0,128^{ε} / 0,560	-0,188^{ε} / 0,389	0,000^{ς} / 1,000	-0,258^{ς} / 0,235
		Phospho(ser65)-PARKIN	0,323^{ς} / 0,133	0,360^{ς} / 0,092	0,353^{ς} / 0,099	-0,235^{ς} / 0,280
		Phospho(ser228)-PINK1	**0,581^{ς} / 0,004**	**0,596^{ς} / 0,003**	**0,613^{ς} / 0,002**	0,016^{ς} / 0,943
		Phospho(ser129)-SNCA	**0,477^{ε} / 0,021**	**0,497^{ε} / 0,016**	**0,604^{ς} / 0,002**	-0,396^{ς} / 0,061
		TH	**0,501^{ε} / 0,015**	**0,462^{ε} / 0,026**	**0,534^{ς} / 0,009**	-0,124^{ς} / 0,572
	Brain cortex	PARKIN	-0,221^{ς} / 0,312	-0,224^{ς} / 0,304	-0,260^{ς} / 0,231	0,016^{ς} / 0,943
		PINK1	0,036^{ε} / 0,871	0,000^{ε} / 0,999	0,006^{ς} / 0,977	-0,133^{ς} / 0,545
		SNCA	0,000^{ς} / 0,998	0,019^{ς} / 0,930	-0,005^{ς} / 0,982	0,102^{ς} / 0,644
		LDLR	-0,140^{ς} / 0,523	-0,223^{ς} / 0,306	-0,210^{ς} / 0,337	0,177^{ς} / 0,420
		Phospho(ser65)-PARKIN	0,030^{ς} / 0,893	0,004^{ς} / 0,986	-0,045^{ς} / 0,837	0,130^{ς} / 0,554
		Phospho(ser228)-PINK1	0,169^{ε} / 0,439	0,116^{ε} / 0,600	0,156^{ς} / 0,478	0,222^{ς} / 0,309
		Phospho(ser129)-SNCA	-0,086^{ε} / 0,695	-0,204^{ε} / 0,351	-0,043^{ς} / 0,846	0,003^{ς} / 0,989
		TH	0,074^{ς} / 0,738	0,086^{ς} / 0,697	-0,063^{ς} / 0,774	0,214^{ς} / 0,326
	Cerebellum	PARKIN	0,007^{ς} / 0,973	-0,077^{ς} / 0,727	0,013^{ς} / 0,954	0,118^{ς} / 0,591
		PINK1	0,102^{ε} / 0,642	0,278^{ε} / 0,199	0,114^{ς} / 0,606	0,250^{ς} / 0,249
		SNCA	0,316^{ς} / 0,142	0,353^{ς} / 0,099	0,301^{ς} / 0,163	0,265^{ς} / 0,222
		LDLR	0,219^{ς} / 0,316	0,204^{ς} / 0,352	0,170^{ς} / 0,438	**0,492^{ς} / 0,017**
		Phospho(ser65)-PARKIN	0,345^{ε} / 0,107	0,162^{ε} / 0,459	0,272^{ς} / 0,210	0,151^{ς} / 0,491
		Phospho(ser228)-PINK1	0,214^{ε} / 0,327	0,319^{ε} / 0,138	0,353^{ς} / 0,099	0,044^{ς} / 0,842
		Phospho(ser129)-SNCA	0,265^{ε} / 0,221	0,149^{ε} / 0,498	0,078^{ς} / 0,723	0,155^{ς} / 0,481
		TH	0,250^{ς} / 0,249	0,241^{ς} / 0,268	0,260^{ς} / 0,231	0,124^{ς} / 0,574
Real Time qPCR	Brain	PARKIN	0,357^{ς} / 0,094	0,248^{ς} / 0,254	0,260^{ς} / 0,231	-0,134 / 0,542
		PINK1	-0,025^{ς} / 0,909	-0,004^{ς} / 0,986	0,039^{ς} / 0,860	0,034 / 0,877
		SNCA	0,265^{ς} / 0,221	0,274^{ς} / 0,206	0,255^{ς} / 0,241	0,161 / 0,464
		LDLR	0,168^{ς} / 0,443	0,044^{ς} / 0,840	0,115^{ς} / 0,601	-0,275 / 0,205
	Cerebellum	PARKIN	0,256^{ς} / 0,239	0,185^{ς} / 0,399	0,210^{ς} / 0,337	0,205^{ς} / 0,349
		PINK1	0,116^{ς} / 0,597	0,201^{ς} / 0,359	0,194^{ς} / 0,375	-0,164^{ς} / 0,454
		SNCA	0,139^{ς} / 0,527	0,077^{ς} / 0,727	0,094^{ς} / 0,668	0,170^{ς} / 0,438
		LDLR	0,392^{ς} / 0,064	0,396^{ς} / 0,061	**0,417^{ς} / 0,048**	0,063^{ς} / 0,774

A value of $p \leq 0.05$ is considered statistically significant and is highlighted in bold characters. ε: Pearson correlation (r) ζ: Spearman's correlation (rs)
