# Supplementary material for: Investigation of correlation between cholesterol intake, apolipoprotein B and Parkinson’s disease related genes in guinea pigs feeding a high-fat diet containing cholesterol
Source: PLoS One. 2026 Jun 25;21(6):e0352642. doi: 10.1371/journal.pone.0352642 (PMC13298788; doi:10.1371/journal.pone.0352642)
Supplement: S12 Table — (PDF) [file pone.0352642.s012.pdf]

S12 Table. Correlation analysis of PARKIN, PINK1, SNCA, LDLR, phospho(ser65)-PARKIN, phospho(ser228)-PINK1, phospho(ser129)-SNCA and TH immunolabelling in the midbrain, brain cortex and cerebellum sections, expression levels of PARKIN, PINK1, SNCA and LDLR genes in the brain and cerebellum tissues and Apo B levels in the serum, brain and cerebellum tissues

n=23			ELISA		
			Apo B		
			Serum	Beyin	Beyincik
			R veya R_s / P	R veya R_s / P	R veya R_s / P
Immunohistochemical Analysis	Midbrain	PARKIN	-0,157$^{\varepsilon}$ / 0,476	0,051ε / 0,817	0,176ε / 0,422
		PINK1	0,221$^{\varepsilon}$ / 0,311	0,274$^{\varepsilon}$ / 0,207	-0,040$^{\varepsilon}$ / 0,856
		SNCA	0,126$^{\varepsilon}$ / 0,565	-0,006$^{\varepsilon}$ / 0,977	-0,150$^{\varepsilon}$ / 0,495
		LDLR	0,080$^{\varepsilon}$ / 0,716	**0,421$^{\varepsilon}$ / 0,045**	0,186$^{\varepsilon}$ / 0,396
		Phospho(ser65)-PARKIN	-0,202$^{\zeta}$ / 0,356	0,201$^{\zeta}$ / 0,359	0,111$^{\zeta}$ / 0,615
		Phospho(ser228)-PINK1	0,167$^{\zeta}$ / 0,446	0,039$^{\zeta}$ / 0,863	-0,321$^{\zeta}$ / 0,136
		Phospho(ser129)-SNCA	-0,186$^{\varepsilon}$ / 0,395	0,125$^{\varepsilon}$ / 0,570	0,174$^{\varepsilon}$ / 0,428
		TH	0,103$^{\varepsilon}$ / 0,641	**0,473$^{\varepsilon}$ / 0,023**	**0,472$^{\varepsilon}$ / 0,023**
	Brain cortex	PARKIN	0,306$^{\zeta}$ / 0,155	0,286ζ / 0,186	-0,011ζ / 0,961
		PINK1	-0,070$^{\varepsilon}$ / 0,751	0,099$^{\varepsilon}$ / 0,654	0,112$^{\varepsilon}$ / 0,611
		SNCA	0,133$^{\zeta}$ / 0,545	0,283$^{\zeta}$ / 0,190	0,071$^{\zeta}$ / 0,746
		LDLR	0,128$^{\zeta}$/ 0,559	0,014$^{\zeta}$ / 0,948	-0,108$^{\zeta}$ / 0,625
		Phospho(ser65)-PARKIN	-0,041$^{\zeta}$ / 0,854	**0,534$^{\zeta}$ / 0,009**	0,026$^{\zeta}$ / 0,906
		Phospho(ser228)-PINK1	0,296$^{\varepsilon}$ / 0,171	0,079$^{\varepsilon}$ / 0,720	-0,172$^{\varepsilon}$ / 0,432
		Phospho(ser129)-SNCA	-0,160$^{\varepsilon}$ / 0,466	-0,037$^{\varepsilon}$ / 0,865	0,194$^{\varepsilon}$ / 0,375
		TH	0,258$^{\zeta}$ / 0,235	0,001$^{\zeta}$ / 0,995	0,190$^{\zeta}$ / 0,385
	Cerebellum	PARKIN	-0,200$^{\zeta}$ / 0,361	-0,005$^{\zeta}$ / 0,980	-0,251$^{\zeta}$ / 0,248
		PINK1	0,085$^{\varepsilon}$ / 0,700	-0,006$^{\varepsilon}$ / 0,979	-0,333$^{\varepsilon}$ / 0,121
		SNCA	0,229$^{\zeta}$ / 0,293	0,188$^{\zeta}$ / 0,390	0,147$^{\zeta}$ / 0,502
		LDLR	0,217$^{\zeta}$ / 0,319	0,105$^{\zeta}$/ 0,634	-0,237$^{\zeta}$ / 0,276
		Phospho(ser65)-PARKIN	-0,246$^{\varepsilon}$ / 0,258	0,043$^{\varepsilon}$ / 0,846	-0,030$^{\varepsilon}$ / 0,890
		Phospho(ser228)-PINK1	0,189$^{\varepsilon}$ / 0,387	0,291$^{\varepsilon}$ / 0,178	-0,104$^{\varepsilon}$ / 0,636
		Phospho(ser129)-SNCA	0,038$^{\varepsilon}$ / 0,864	0,145$^{\varepsilon}$ / 0,508	0,058$^{\varepsilon}$ / 0,793
		TH	-0,036$^{\zeta}$ / 0,872	-0,019$^{\zeta}$ / 0,930	0,107$^{\zeta}$ / 0,626
Real Time qPCR	Brain	PARKIN	0,362$^{\zeta}$ / 0,090	0,288$^{\zeta}$ / 0,182	0,385$^{\zeta}$ / 0,069
		PINK1	-0,115$^{\zeta}$ / 0,603	-0,121$^{\zeta}$ / 0,582	-0,402$^{\zeta}$ / 0,057
		SNCA	0,328$^{\zeta}$ / 0,126	0,388$^{\zeta}$ / 0,067	0,216$^{\zeta}$ / 0,321
		LDLR	-0,050$^{\zeta}$/ 0,819	0,003$^{\zeta}$ / 0,988	0,172$^{\zeta}$ / 0,431
	Cerebellum	PARKIN	0,281$^{\zeta}$ / 0,195	-0,037$^{\zeta}$ / 0,867	0,064$^{\zeta}$ / 0,773
		PINK1	-0,279$^{\zeta}$ / 0,198	0,094$^{\zeta}$ / 0,668	-0,024$^{\zeta}$ / 0,913
		SNCA	0,142$^{\zeta}$ / 0,517	-0,002$^{\zeta}$ / 0,993	-0,149$^{\zeta}$ / 0,497
		LDLR	-0,095$^{\zeta}$ / 0,667	0,194$^{\zeta}$ / 0,375	0,225$^{\zeta}$ / 0,301

A value of $p \leq 0.05$ is considered statistically significant and is highlighted in bold characters. ε: Pearson correlation (r) ζ: Spearman's correlation (rs)
