# Supplementary material for: Investigation of correlation between cholesterol intake, apolipoprotein B and Parkinson’s disease related genes in guinea pigs feeding a high-fat diet containing cholesterol
Source: PLoS One. 2026 Jun 25;21(6):e0352642. doi: 10.1371/journal.pone.0352642 (PMC13298788; doi:10.1371/journal.pone.0352642)
Supplement: S13. Table — (PDF) [file pone.0352642.s013.pdf]

S13 Table. Correlation analysis of CHOL, LDL, HDL and GLU levels in the serum and Apo B levels in the serum, brain and cerebellum tissues									
n=23			**ELISA**			**Biochemical Analysis**			
			**Apo B**			**Serum**			
			**Serum**	**Brain**	**Cerebellum**	**CHOL**	**LDL**	**HDL**	**GLU**
			**R veya Rs / P**	**R veya Rs / P**	**R veya Rs / P**	**R veya Rs / P**	**R veya Rs / P**	**R veya Rs / P**	**R veya Rs / P**
**Biochemical Analysis**	**Serum**	**CHOL**	0,031$^{\varepsilon}$ / 0,888	0,138$^{\varepsilon}$ / 0,529	-0,013$^{\varepsilon}$ / 0,952	-	**0,925$^{\varepsilon}$ / 0,000**	**0,960$^{\zeta}$ / 0,000**	0,230$^{\zeta}$ / 0,291
		**LDL**	0,132$^{\varepsilon}$ / 0,547	0,119$^{\varepsilon}$ / 0,589	-0,152$^{\varepsilon}$ / 0,488	**0,925$^{\varepsilon}$ / 0,000**	-	**0,946$^{\zeta}$ / 0,000**	0,137$^{\zeta}$ / 0,533
		**HDL**	0,110$^{\zeta}$ / 0,618	0,125$^{\zeta}$ / 0,571	-0,051$^{\zeta}$ / 0,818	**0,960$^{\zeta}$ / 0,000**	**0,946$^{\zeta}$ / 0,000**	-	0,154$^{\zeta}$ / 0,482
		**GLU**	0,262$^{\zeta}$ / 0,228	-0,119$^{\zeta}$ / 0,589	**-0,515$^{\zeta}$ / 0,012**	0,230$^{\zeta}$ / 0,291	0,137$^{\zeta}$ / 0,533	0,154$^{\zeta}$ / 0,482	-
**ELISA**	**Apo B**	**Serum**	-	0,248$^{\varepsilon}$ / 0,254	-0,032$^{\varepsilon}$ / 0,886	0,031$^{\varepsilon}$ / 0,888	0,132$^{\varepsilon}$ / 0,547	0,110$^{\zeta}$ / 0,618	0,262$^{\zeta}$ / 0,228
		**Brain**	0,248$^{\varepsilon}$ / 0,254	-	**0,420$^{\varepsilon}$ / 0,046**	0,138$^{\varepsilon}$ / 0,529	0,119$^{\varepsilon}$ / 0,589	0,125$^{\zeta}$ / 0,571	-0,119$^{\zeta}$ / 0,589
		**Cerebellum**	-0,032$^{\varepsilon}$ / 0,886	**0,420$^{\varepsilon}$ / 0,046**	-	-0,013$^{\varepsilon}$ / 0,952	-0,152$^{\varepsilon}$ / 0,488	-0,051$^{\zeta}$ / 0,818	**-0,515$^{\zeta}$ / 0,012**

A value of $p \leq 0.05$ is considered statistically significant and is highlighted in bold characters. ε: Pearson correlation (r) ζ: Spearman's correlation (rs)
